# Supplementary material for: The natural history of symptomatic COVID-19 during the first wave in Catalonia
Source: Nat Commun. 2021 Feb 3;12:777. doi: 10.1038/s41467-021-21100-y (PMC7858639; doi:10.1038/s41467-021-21100-y)

Appendix

Contents

Appendix ... 1

Figure 1. Study inclusion flow chart ... 1

Figure 2. Log-log plot for age and risk of transition from general population to diagnosis with COVID-19 ... 2

Table 1. Estimated hazard ratios for age: from general population to diagnosed with COVID-19, overall and stratified by age ... 3

Table 2. Estimated hazard ratios for age: from general population to diagnosed with COVID-19, stratified by calendar month ... 4

Table 3. Estimated hazard ratios for age: from general population to diagnosed with COVID-19, stratified by region ... 5

Table 4. Estimated hazard ratios for age: from general population to hospitalised with COVID-19, overall and stratified by age ... 6

Table 5. Estimated hazard ratios for age: from general population to hospitalised with COVID-19, stratified by calendar month ... 7

Table 6. Estimated hazard ratios for age: from general population to hospitalised with COVID-19, stratified by region ... 8

Table 7. Estimated hazard ratios for age: from diagnosed with COVID-19 to hospitalised with COVID-19, overall and stratified by age ... 9

Table 8. Estimated hazard ratios for age: from diagnosed with COVID-19 to hospitalised with COVID-19, stratified by calendar month ... 10

Table 9. Estimated hazard ratios for age: from diagnosed with COVID-19 to hospitalised with COVID-19, stratified by region ... 11

Table 10. Estimated hazard ratios for age: from diagnosed with COVID-19 to death, overall and stratified by age ... 12

Table 11. Estimated hazard ratios for age: from diagnosed with COVID-19 to death, stratified by calendar month ... 13

Table 12. Estimated hazard ratios for age: from diagnosed with COVID-19 to death, stratified by region ... 14

Table 13. Estimated hazard ratios for age: from hospitalised with COVID-19 to death, overall and stratified by age ... 16

Table 14. Estimated hazard ratios for age: from hospitalised with COVID-19 to death, stratified by calendar month ... 17

Table 15. Estimated hazard ratios for age: from hospitalised with COVID-19 to death, stratified by region ... 18

Table 16. Estimated hazard ratios for male sex: overall and stratified by age ... 1

Table 17. Estimated hazard ratios for male sex: stratified by calendar month 2
Table 18. Estimated hazard ratios for male sex: stratified by region 3
Table 19. Estimated hazard ratios for comorbidities: overall and stratified by age 4
Table 20. Estimated hazard ratios for comorbidities: stratified by calendar month 9
Figure 3. Comorbidities and COVID-19 transitions: stratified by calendar month (adjusted for age and sex) 12
Table 21. Estimated hazard ratios for comorbidities: stratified by region 13
Figure 4. Comorbidities and COVID-19 transitions: stratified by region (adjusted for age and sex) 17

Figure 1. Study inclusion flow chart

A flow chart describing entry into the study.

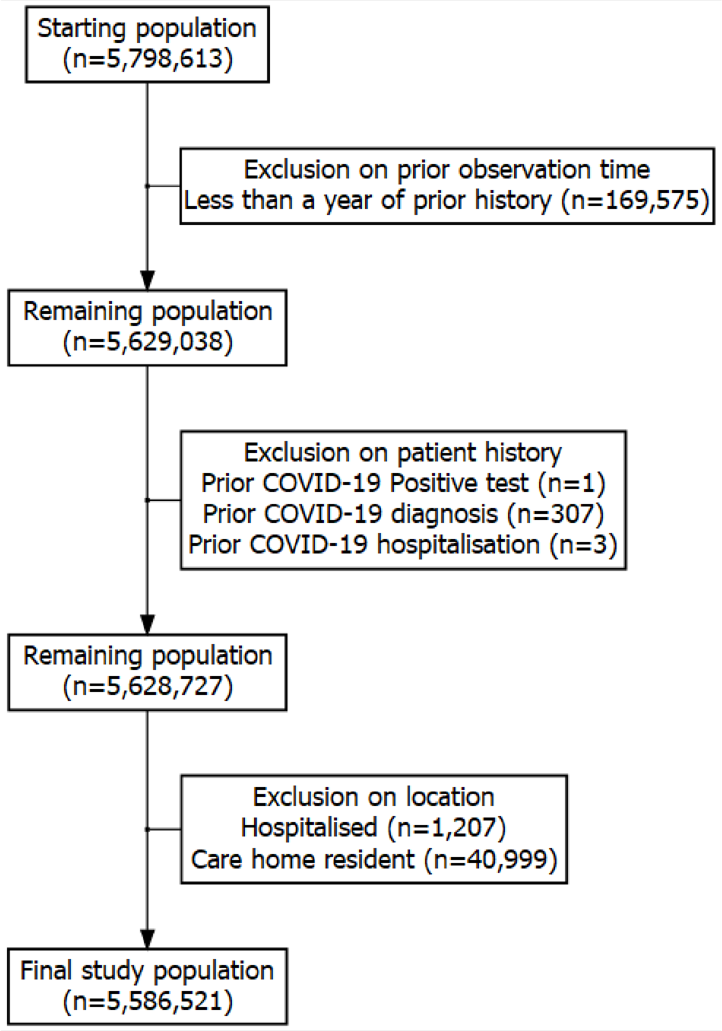

Figure 2. Log-log plot for age and risk of transition from general population to diagnosis with COVID-19

A log-log plot to assess non-proportionality in hazards.

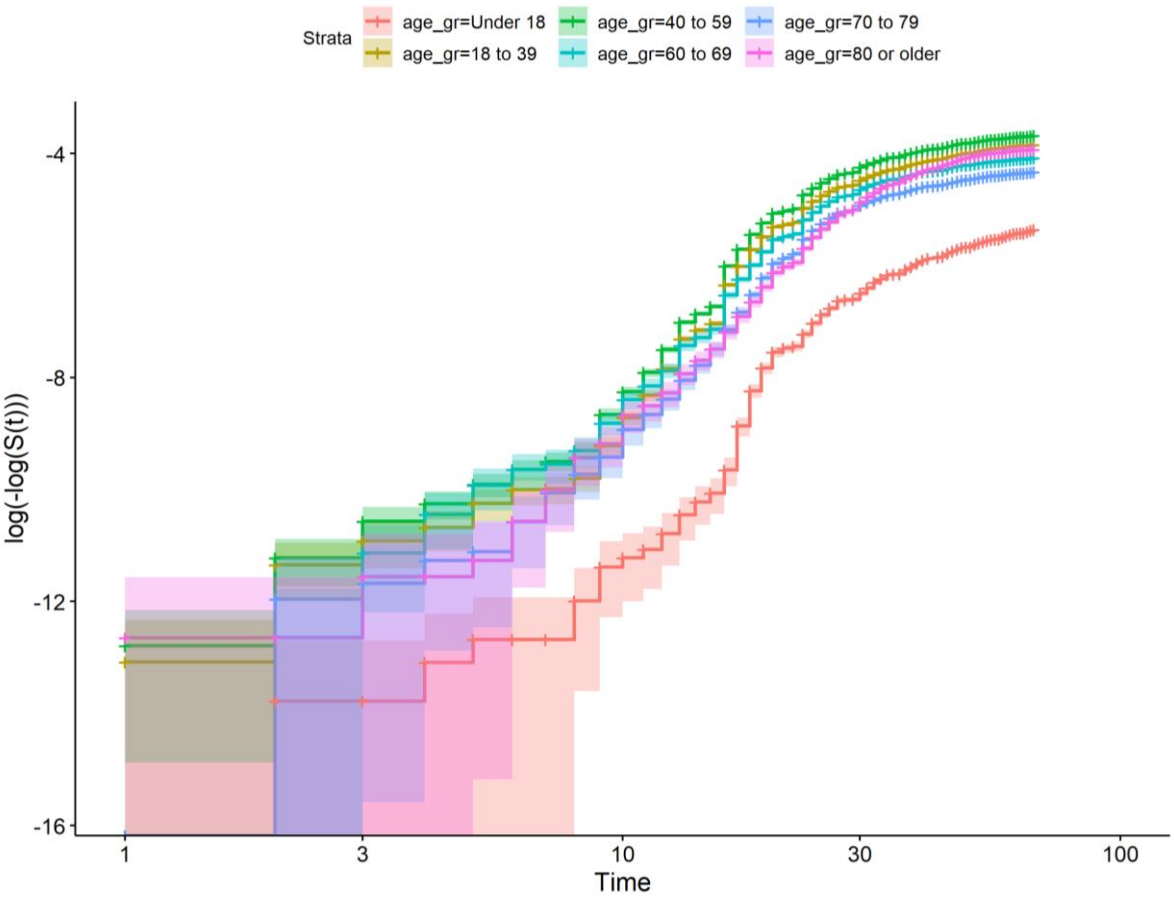

Table 1. Estimated hazard ratios for age: from general population to diagnosed with COVID-19, overall and stratified by age

From general population to diagnosed with COVID-19						
**Relative hazard ratios (95% CI) for age from models**						
	**Overall**		**Male**		**Female**	
**Age**	**Age only model**	**Age and comorbidity model**	**Age only model**	**Age and comorbidity model**	**Age only model**	**Age and comorbidity model**
20	0.70 (0.69 to 0.72)	0.81 (0.80 to 0.83)	0.68 (0.66 to 0.70)	0.80 (0.77 to 0.82)	0.73 (0.71 to 0.75)	0.84 (0.82 to 0.86)
25	0.95 (0.93 to 0.97)	1.09 (1.07 to 1.12)	0.87 (0.85 to 0.90)	1.03 (0.99 to 1.06)	1.02 (0.99 to 1.04)	1.17 (1.14 to 1.20)
30	1.22 (1.20 to 1.24)	1.40 (1.37 to 1.42)	1.10 (1.07 to 1.13)	1.28 (1.24 to 1.33)	1.34 (1.31 to 1.37)	1.53 (1.49 to 1.57)
35	1.46 (1.43 to 1.49)	1.66 (1.62 to 1.69)	1.31 (1.27 to 1.34)	1.52 (1.47 to 1.57)	1.62 (1.58 to 1.66)	1.83 (1.79 to 1.88)
40	1.60 (1.57 to 1.63)	1.80 (1.76 to 1.84)	1.44 (1.40 to 1.48)	1.65 (1.60 to 1.71)	1.78 (1.74 to 1.83)	2.00 (1.95 to 2.06)
45	1.57 (1.55 to 1.60)	1.75 (1.72 to 1.79)	1.42 (1.38 to 1.46)	1.61 (1.56 to 1.66)	1.75 (1.71 to 1.80)	1.95 (1.90 to 2.00)
50	1.41 (1.39 to 1.43)	1.55 (1.53 to 1.57)	1.27 (1.25 to 1.30)	1.41 (1.38 to 1.44)	1.55 (1.52 to 1.58)	1.71 (1.68 to 1.75)
55	1.22 (1.21 to 1.23)	1.31 (1.30 to 1.32)	1.12 (1.11 to 1.13)	1.21 (1.19 to 1.22)	1.31 (1.29 to 1.32)	1.42 (1.40 to 1.43)
60	1.07 (1.07 to 1.08)	1.12 (1.12 to 1.13)	1.03 (1.02 to 1.03)	1.07 (1.06 to 1.08)	1.11 (1.11 to 1.12)	1.17 (1.16 to 1.18)
65	ref	ref	ref	ref	ref	ref
70	0.97 (0.97 to 0.97)	0.92 (0.91 to 0.92)	1.01 (1.01 to 1.02)	0.97 (0.96 to 0.98)	0.94 (0.93 to 0.94)	0.88 (0.88 to 0.89)
75	0.97 (0.96 to 0.98)	0.86 (0.85 to 0.88)	1.05 (1.04 to 1.07)	0.96 (0.94 to 0.98)	0.91 (0.90 to 0.92)	0.80 (0.78 to 0.81)
80	0.98 (0.96 to 1.00)	0.82 (0.80 to 0.84)	1.11 (1.07 to 1.14)	0.95 (0.92 to 0.99)	0.90 (0.88 to 0.92)	0.73 (0.71 to 0.75)
85	1.00 (0.97 to 1.02)	0.78 (0.76 to 0.81)	1.16 (1.12 to 1.21)	0.95 (0.91 to 1.00)	0.90 (0.87 to 0.93)	0.67 (0.64 to 0.70)
90	1.02 (0.98 to 1.05)	0.75 (0.72 to 0.78)	1.22 (1.16 to 1.28)	0.95 (0.90 to 1.01)	0.90 (0.86 to 0.94)	0.62 (0.58 to 0.65)
95	1.03 (0.99 to 1.08)	0.71 (0.68 to 0.75)	1.28 (1.20 to 1.36)	0.95 (0.89 to 1.02)	0.90 (0.85 to 0.95)	0.57 (0.53 to 0.60)

Table 2. Estimated hazard ratios for age: from general population to diagnosed with COVID-19, stratified by calendar month

**From diagnosed with COVID-19 to hospitalised with COVID-19**				
**Relative hazard ratios (95% CI) for age from models**				
	**March**		**April**	
**Age**	**Age only model**	**Age and comorbidity model**	**Age only model**	**Age and comorbidity model**
20	0.57 (0.55 to 0.58)	0.64 (0.62 to 0.66)	0.89 (0.86 to 0.91)	1.05 (1.02 to 1.09)
25	0.84 (0.82 to 0.86)	0.95 (0.92 to 0.97)	1.12 (1.08 to 1.15)	1.32 (1.28 to 1.37)
30	1.14 (1.11 to 1.16)	1.27 (1.24 to 1.31)	1.37 (1.33 to 1.41)	1.61 (1.56 to 1.67)
35	1.38 (1.35 to 1.41)	1.53 (1.49 to 1.57)	1.60 (1.55 to 1.64)	1.86 (1.80 to 1.92)
40	1.52 (1.48 to 1.55)	1.67 (1.63 to 1.72)	1.71 (1.66 to 1.76)	1.97 (1.91 to 2.04)
45	1.52 (1.48 to 1.55)	1.66 (1.61 to 1.70)	1.63 (1.58 to 1.68)	1.85 (1.80 to 1.91)
50	1.41 (1.38 to 1.43)	1.52 (1.49 to 1.55)	1.40 (1.37 to 1.43)	1.57 (1.53 to 1.60)
55	1.25 (1.24 to 1.27)	1.33 (1.31 to 1.35)	1.17 (1.15 to 1.18)	1.28 (1.26 to 1.30)
60	1.11 (1.11 to 1.12)	1.15 (1.14 to 1.16)	1.03 (1.03 to 1.04)	1.09 (1.08 to 1.10)
65	1.00 (1.00 to 1.00)	1.00 (1.00 to 1.00)	1.00 (1.00 to 1.00)	1.00 (1.00 to 1.00)
70	0.91 (0.90 to 0.92)	0.87 (0.87 to 0.88)	1.04 (1.04 to 1.05)	0.98 (0.97 to 0.98)
75	0.83 (0.82 to 0.85)	0.77 (0.75 to 0.78)	1.15 (1.13 to 1.17)	0.99 (0.97 to 1.01)
80	0.77 (0.75 to 0.79)	0.67 (0.65 to 0.69)	1.30 (1.27 to 1.33)	1.03 (1.00 to 1.07)
85	0.71 (0.68 to 0.73)	0.59 (0.57 to 0.62)	1.48 (1.43 to 1.53)	1.08 (1.04 to 1.13)
90	0.65 (0.62 to 0.68)	0.52 (0.49 to 0.55)	1.68 (1.61 to 1.76)	1.13 (1.07 to 1.20)
95	0.60 (0.56 to 0.64)	0.46 (0.43 to 0.49)	1.92 (1.81 to 2.03)	1.18 (1.11 to 1.27)

Table 3. Estimated hazard ratios for age: from general population to diagnosed with COVID-19, stratified by region

From general population to diagnosed with COVID-19								
**Relative hazard ratios (95% CI) for age from models**								
	**Barcelona**		**Girona**		**Lleida**		**Tarragona**	
**Age**	**Age only model**	**Age and comorbidity model**	**Age only model**	**Age and comorbidity model**	**Age only model**	**Age and comorbidity model**	**Age only model**	**Age and comorbidity model**
20	0.64 (0.63 to 0.65)	0.73 (0.71 to 0.75)	0.90 (0.85 to 0.96)	1.06 (0.99 to 1.13)	1.00 (0.93 to 1.08)	1.20 (1.10 to 1.32)	1.07 (0.97 to 1.18)	1.28 (1.15 to 1.42)
25	0.90 (0.88 to 0.92)	1.03 (1.01 to 1.05)	1.12 (1.05 to 1.19)	1.30 (1.22 to 1.39)	1.13 (1.04 to 1.22)	1.34 (1.22 to 1.47)	1.40 (1.28 to 1.54)	1.67 (1.50 to 1.87)
30	1.18 (1.16 to 1.20)	1.34 (1.31 to 1.37)	1.40 (1.32 to 1.49)	1.62 (1.52 to 1.74)	1.34 (1.24 to 1.45)	1.59 (1.45 to 1.74)	1.70 (1.55 to 1.87)	2.02 (1.81 to 2.26)
35	1.41 (1.38 to 1.44)	1.59 (1.55 to 1.62)	1.71 (1.61 to 1.81)	1.96 (1.84 to 2.10)	1.63 (1.51 to 1.77)	1.92 (1.76 to 2.11)	1.90 (1.72 to 2.09)	2.24 (2.01 to 2.50)
40	1.54 (1.51 to 1.57)	1.71 (1.68 to 1.75)	1.90 (1.79 to 2.02)	2.16 (2.02 to 2.32)	1.88 (1.73 to 2.05)	2.19 (2.00 to 2.41)	1.93 (1.74 to 2.13)	2.25 (2.01 to 2.52)
45	1.52 (1.49 to 1.55)	1.68 (1.64 to 1.71)	1.82 (1.72 to 1.92)	2.05 (1.93 to 2.18)	1.90 (1.75 to 2.06)	2.18 (2.00 to 2.39)	1.78 (1.61 to 1.96)	2.05 (1.85 to 2.28)
50	1.38 (1.35 to 1.40)	1.50 (1.48 to 1.53)	1.53 (1.47 to 1.59)	1.71 (1.63 to 1.78)	1.64 (1.55 to 1.74)	1.85 (1.73 to 1.97)	1.51 (1.41 to 1.63)	1.72 (1.58 to 1.86)
55	1.20 (1.19 to 1.21)	1.29 (1.28 to 1.30)	1.25 (1.22 to 1.28)	1.36 (1.33 to 1.40)	1.32 (1.28 to 1.37)	1.45 (1.39 to 1.50)	1.26 (1.21 to 1.31)	1.39 (1.32 to 1.45)
60	1.07 (1.07 to 1.08)	1.12 (1.11 to 1.12)	1.07 (1.06 to 1.08)	1.13 (1.11 to 1.15)	1.10 (1.09 to 1.12)	1.16 (1.14 to 1.18)	1.09 (1.07 to 1.10)	1.15 (1.12 to 1.17)
65	1.00 (1.00 to 1.00)	1.00 (1.00 to 1.00)	1.00 (1.00 to 1.00)	1.00 (1.00 to 1.00)	1.00 (1.00 to 1.00)	1.00 (1.00 to 1.00)	1.00 (1.00 to 1.00)	1.00 (1.00 to 1.00)
70	0.97 (0.96 to 0.97)	0.92 (0.91 to 0.93)	0.99 (0.97 to 1.01)	0.93 (0.91 to 0.95)	0.97 (0.95 to 0.99)	0.92 (0.90 to 0.94)	0.97 (0.95 to 1.00)	0.91 (0.88 to 0.94)
75	0.96 (0.95 to 0.97)	0.86 (0.85 to 0.88)	1.02 (0.98 to 1.06)	0.89 (0.85 to 0.93)	0.99 (0.95 to 1.04)	0.88 (0.83 to 0.93)	0.98 (0.92 to 1.04)	0.85 (0.79 to 0.91)
80	0.97 (0.95 to 0.99)	0.82 (0.80 to 0.84)	1.07 (1.00 to 1.13)	0.86 (0.81 to 0.93)	1.04 (0.96 to 1.12)	0.86 (0.79 to 0.94)	1.00 (0.91 to 1.11)	0.80 (0.72 to 0.90)
85	0.98 (0.95 to 1.01)	0.78 (0.75 to 0.80)	1.12 (1.03 to 1.21)	0.84 (0.76 to 0.93)	1.10 (0.99 to 1.22)	0.85 (0.75 to 0.97)	1.04 (0.90 to 1.19)	0.77 (0.65 to 0.90)
90	0.99 (0.95 to 1.02)	0.74 (0.71 to 0.77)	1.17 (1.05 to 1.30)	0.82 (0.72 to 0.93)	1.16 (1.01 to 1.34)	0.84 (0.71 to 0.99)	1.07 (0.89 to 1.28)	0.73 (0.59 to 0.89)
95	1.00 (0.95 to 1.04)	0.70 (0.66 to 0.74)	1.23 (1.08 to 1.40)	0.80 (0.68 to 0.93)	1.23 (1.03 to 1.46)	0.83 (0.68 to 1.02)	1.10 (0.88 to 1.37)	0.69 (0.54 to 0.89)

Table 4. Estimated hazard ratios for age: from general population to hospitalised with COVID-19, overall and stratified by age

**From general population to hospitalised with COVID-19**						
**Relative hazard ratios (95% CI) for age from models**						
	**Overall**		**Male**		**Female**	
**Age**	**Age only model**	**Age and comorbidity model**	**Age only model**	**Age and comorbidity model**	**Age only model**	**Age and comorbidity model**
20	0.038 (0.033 to 0.043)	0.064 (0.056 to 0.073)	0.026 (0.022 to 0.032)	0.044 (0.036 to 0.053)	0.058 (0.049 to 0.07)	0.097 (0.081 to 0.12)
25	0.059 (0.053 to 0.066)	0.097 (0.087 to 0.11)	0.043 (0.037 to 0.05)	0.069 (0.059 to 0.08)	0.085 (0.073 to 0.098)	0.14 (0.12 to 0.16)
30	0.091 (0.084 to 0.099)	0.14 (0.13 to 0.15)	0.07 (0.062 to 0.079)	0.11 (0.093 to 0.12)	0.12 (0.11 to 0.14)	0.19 (0.17 to 0.21)
35	0.14 (0.13 to 0.15)	0.20 (0.19 to 0.22)	0.11 (0.10 to 0.12)	0.16 (0.14 to 0.17)	0.17 (0.16 to 0.19)	0.25 (0.23 to 0.28)
40	0.20 (0.19 to 0.21)	0.28 (0.27 to 0.30)	0.17 (0.16 to 0.18)	0.23 (0.21 to 0.25)	0.24 (0.22 to 0.25)	0.33 (0.31 to 0.36)
45	0.29 (0.28 to 0.30)	0.38 (0.37 to 0.40)	0.25 (0.24 to 0.26)	0.32 (0.31 to 0.34)	0.33 (0.31 to 0.34)	0.43 (0.41 to 0.46)
50	0.41 (0.40 to 0.42)	0.51 (0.49 to 0.52)	0.37 (0.36 to 0.38)	0.45 (0.43 to 0.46)	0.44 (0.43 to 0.46)	0.55 (0.53 to 0.57)
55	0.56 (0.55 to 0.57)	0.65 (0.64 to 0.66)	0.53 (0.52 to 0.54)	0.60 (0.59 to 0.61)	0.59 (0.58 to 0.60)	0.68 (0.67 to 0.70)
60	0.76 (0.75 to 0.76)	0.82 (0.81 to 0.82)	0.73 (0.73 to 0.74)	0.79 (0.78 to 0.79)	0.77 (0.77 to 0.78)	0.84 (0.82 to 0.85)
65	ref	ref	ref	ref	ref	ref
70	1.29 (1.28 to 1.30)	1.19 (1.18 to 1.20)	1.33 (1.32 to 1.34)	1.24 (1.22 to 1.26)	1.27 (1.26 to 1.29)	1.17 (1.16 to 1.19)
75	1.63 (1.61 to 1.66)	1.38 (1.36 to 1.41)	1.73 (1.69 to 1.77)	1.50 (1.46 to 1.54)	1.60 (1.56 to 1.64)	1.35 (1.31 to 1.39)
80	2.02 (1.97 to 2.08)	1.57 (1.51 to 1.62)	2.19 (2.11 to 2.27)	1.76 (1.68 to 1.85)	1.98 (1.90 to 2.06)	1.53 (1.45 to 1.60)
85	2.45 (2.35 to 2.55)	1.73 (1.64 to 1.82)	2.71 (2.56 to 2.87)	2.02 (1.88 to 2.16)	2.41 (2.28 to 2.56)	1.69 (1.57 to 1.82)
90	2.91 (2.75 to 3.08)	1.86 (1.73 to 1.99)	3.27 (3.02 to 3.55)	2.25 (2.04 to 2.47)	2.90 (2.67 to 3.14)	1.83 (1.66 to 2.03)
95	3.37 (3.13 to 3.64)	1.94 (1.78 to 2.13)	3.86 (3.47 to 4.30)	2.44 (2.15 to 2.77)	3.43 (3.08 to 3.82)	1.95 (1.71 to 2.22)

Table 5. Estimated hazard ratios for age: from general population to hospitalised with COVID-19, stratified by calendar month

From general population to hospitalised with COVID-19				
**Relative hazard ratios (95% CI) for age from models**				
	**March**		**April**	
**Age**	**Age only model**	**Age and comorbidity model**	**Age only model**	**Age and comorbidity model**
20	0.035 (0.029 to 0.043)	0.053 (0.043 to 0.065)	0.065 (0.052 to 0.083)	0.10 (0.078 to 0.13)
25	0.062 (0.052 to 0.073)	0.091 (0.076 to 0.11)	0.10 (0.083 to 0.13)	0.16 (0.12 to 0.20)
30	0.09 (0.077 to 0.10)	0.13 (0.11 to 0.16)	0.15 (0.13 to 0.19)	0.24 (0.19 to 0.29)
35	0.11 (0.10 to 0.13)	0.17 (0.15 to 0.19)	0.22 (0.19 to 0.26)	0.34 (0.29 to 0.40)
40	0.14 (0.13 to 0.15)	0.21 (0.19 to 0.23)	0.30 (0.26 to 0.34)	0.46 (0.40 to 0.53)
45	0.19 (0.17 to 0.21)	0.28 (0.25 to 0.30)	0.39 (0.34 to 0.44)	0.57 (0.50 to 0.66)
50	0.30 (0.27 to 0.32)	0.40 (0.37 to 0.43)	0.49 (0.44 to 0.54)	0.67 (0.60 to 0.75)
55	0.48 (0.46 to 0.50)	0.58 (0.55 to 0.61)	0.61 (0.58 to 0.65)	0.76 (0.71 to 0.81)
60	0.73 (0.72 to 0.74)	0.80 (0.78 to 0.81)	0.77 (0.76 to 0.79)	0.86 (0.84 to 0.88)
65	1.00 (1.00 to 1.00)	1.00 (1.00 to 1.00)	1.00 (1.00 to 1.00)	1.00 (1.00 to 1.00)
70	1.25 (1.24 to 1.27)	1.17 (1.15 to 1.18)	1.32 (1.30 to 1.34)	1.18 (1.16 to 1.21)
75	1.48 (1.45 to 1.52)	1.29 (1.25 to 1.33)	1.76 (1.70 to 1.82)	1.42 (1.36 to 1.48)
80	1.70 (1.63 to 1.77)	1.39 (1.32 to 1.47)	2.37 (2.23 to 2.51)	1.72 (1.60 to 1.84)
85	1.93 (1.81 to 2.05)	1.49 (1.39 to 1.61)	3.19 (2.93 to 3.47)	2.08 (1.87 to 2.30)
90	2.19 (2.02 to 2.38)	1.60 (1.45 to 1.76)	4.30 (3.84 to 4.81)	2.51 (2.20 to 2.87)
95	2.49 (2.25 to 2.75)	1.72 (1.52 to 1.93)	5.79 (5.04 to 6.65)	3.04 (2.58 to 3.58)

Table 6. Estimated hazard ratios for age: from general population to hospitalised with COVID-19, stratified by region

From general population to hospitalised with COVID-19								
**Relative hazard ratios (95% CI) for age from models**								
	**Barcelona**		**Girona**		**Lleida**		**Tarragona**	
**Age**	**Age only model**	**Age and comorbidity model**	**Age only model**	**Age and comorbidity model**	**Age only model**	**Age and comorbidity model**	**Age only model**	**Age and comorbidity model**
20	0.033 (0.028 to 0.038)	0.056 (0.049 to 0.065)	0.10 (0.07 to 0.15)	0.17 (0.12 to 0.25)	0.044 (0.02 to 0.097)	0.086 (0.039 to 0.19)	0.068 (0.034 to 0.14)	0.10 (0.051 to 0.21)
25	0.053 (0.047 to 0.059)	0.087 (0.077 to 0.098)	0.13 (0.096 to 0.17)	0.21 (0.15 to 0.29)	0.066 (0.035 to 0.12)	0.12 (0.063 to 0.22)	0.093 (0.053 to 0.16)	0.14 (0.077 to 0.24)
30	0.083 (0.076 to 0.091)	0.13 (0.12 to 0.14)	0.16 (0.13 to 0.21)	0.26 (0.20 to 0.33)	0.096 (0.058 to 0.16)	0.16 (0.097 to 0.27)	0.13 (0.082 to 0.20)	0.18 (0.11 to 0.29)
35	0.13 (0.12 to 0.14)	0.19 (0.18 to 0.21)	0.21 (0.18 to 0.25)	0.31 (0.25 to 0.39)	0.14 (0.094 to 0.20)	0.22 (0.15 to 0.32)	0.17 (0.12 to 0.24)	0.24 (0.16 to 0.34)
40	0.19 (0.18 to 0.20)	0.27 (0.26 to 0.29)	0.27 (0.24 to 0.31)	0.38 (0.32 to 0.45)	0.20 (0.15 to 0.26)	0.29 (0.21 to 0.39)	0.23 (0.18 to 0.30)	0.31 (0.23 to 0.41)
45	0.28 (0.27 to 0.29)	0.37 (0.36 to 0.39)	0.35 (0.31 to 0.39)	0.47 (0.41 to 0.53)	0.28 (0.23 to 0.35)	0.38 (0.30 to 0.48)	0.31 (0.26 to 0.37)	0.40 (0.32 to 0.49)
50	0.40 (0.39 to 0.41)	0.50 (0.48 to 0.51)	0.45 (0.42 to 0.49)	0.57 (0.51 to 0.62)	0.39 (0.34 to 0.45)	0.49 (0.42 to 0.58)	0.42 (0.37 to 0.48)	0.50 (0.43 to 0.59)
55	0.56 (0.55 to 0.57)	0.65 (0.63 to 0.66)	0.59 (0.56 to 0.62)	0.69 (0.64 to 0.73)	0.54 (0.50 to 0.59)	0.63 (0.57 to 0.70)	0.56 (0.52 to 0.61)	0.64 (0.58 to 0.71)
60	0.76 (0.75 to 0.76)	0.82 (0.81 to 0.82)	0.76 (0.75 to 0.78)	0.83 (0.80 to 0.86)	0.74 (0.71 to 0.77)	0.80 (0.76 to 0.84)	0.75 (0.72 to 0.78)	0.80 (0.76 to 0.84)
65	1.00 (1.00 to 1.00)	1.00 (1.00 to 1.00)	1.00 (1.00 to 1.00)	1.00 (1.00 to 1.00)	1.00 (1.00 to 1.00)	1.00 (1.00 to 1.00)	1.00 (1.00 to 1.00)	1.00 (1.00 to 1.00)
70	1.29 (1.28 to 1.30)	1.19 (1.18 to 1.20)	1.31 (1.27 to 1.35)	1.20 (1.16 to 1.25)	1.34 (1.28 to 1.39)	1.23 (1.17 to 1.30)	1.33 (1.27 to 1.39)	1.24 (1.17 to 1.31)
75	1.62 (1.59 to 1.65)	1.38 (1.35 to 1.41)	1.73 (1.62 to 1.84)	1.45 (1.32 to 1.58)	1.76 (1.61 to 1.93)	1.50 (1.34 to 1.69)	1.76 (1.60 to 1.94)	1.52 (1.34 to 1.71)
80	1.99 (1.93 to 2.04)	1.55 (1.49 to 1.61)	2.28 (2.04 to 2.54)	1.73 (1.50 to 2.00)	2.30 (1.97 to 2.68)	1.81 (1.49 to 2.19)	2.33 (1.98 to 2.73)	1.85 (1.51 to 2.25)
85	2.37 (2.27 to 2.48)	1.69 (1.60 to 1.78)	3.02 (2.57 to 3.55)	2.07 (1.68 to 2.54)	2.97 (2.36 to 3.72)	2.15 (1.62 to 2.84)	3.06 (2.42 to 3.88)	2.23 (1.67 to 2.97)
90	2.77 (2.60 to 2.94)	1.79 (1.67 to 1.93)	4.01 (3.21 to 5.02)	2.47 (1.87 to 3.26)	3.78 (2.75 to 5.20)	2.52 (1.72 to 3.69)	4.03 (2.90 to 5.59)	2.67 (1.80 to 3.95)
95	3.14 (2.90 to 3.41)	1.85 (1.68 to 2.04)	5.35 (4.00 to 7.18)	2.94 (2.05 to 4.21)	4.77 (3.12 to 7.29)	2.92 (1.77 to 4.82)	5.28 (3.41 to 8.15)	3.17 (1.90 to 5.30)

Table 7. Estimated hazard ratios for age: from diagnosed with COVID-19 to hospitalised with COVID-19, overall and stratified by age

From diagnosed with COVID-19 to hospitalised with COVID-19						
**Relative hazard ratios (95% CI) for age from models**						
	**Overall**		**Male**		**Female**	
**Age**	**Age only model**	**Age and comorbidity model**	**Age only model**	**Age and comorbidity model**	**Age only model**	**Age and comorbidity model**
20	0.076 (0.067 to 0.087)	0.092 (0.081 to 0.11)	0.054 (0.044 to 0.065)	0.061 (0.05 to 0.074)	0.10 (0.088 to 0.12)	0.13 (0.11 to 0.15)
25	0.10 (0.092 to 0.11)	0.12 (0.11 to 0.13)	0.075 (0.066 to 0.086)	0.085 (0.074 to 0.098)	0.13 (0.11 to 0.15)	0.16 (0.14 to 0.18)
30	0.13 (0.12 to 0.14)	0.15 (0.14 to 0.17)	0.10 (0.094 to 0.12)	0.12 (0.10 to 0.13)	0.16 (0.14 to 0.18)	0.18 (0.16 to 0.21)
35	0.16 (0.15 to 0.17)	0.19 (0.18 to 0.21)	0.14 (0.13 to 0.16)	0.16 (0.14 to 0.18)	0.18 (0.16 to 0.20)	0.21 (0.19 to 0.24)
40	0.19 (0.18 to 0.20)	0.23 (0.21 to 0.25)	0.19 (0.18 to 0.21)	0.22 (0.20 to 0.24)	0.20 (0.18 to 0.21)	0.23 (0.21 to 0.26)
45	0.24 (0.23 to 0.26)	0.29 (0.27 to 0.31)	0.27 (0.24 to 0.29)	0.30 (0.27 to 0.33)	0.22 (0.20 to 0.24)	0.27 (0.24 to 0.30)
50	0.36 (0.34 to 0.38)	0.41 (0.38 to 0.43)	0.40 (0.37 to 0.43)	0.43 (0.40 to 0.47)	0.32 (0.29 to 0.35)	0.37 (0.34 to 0.41)
55	0.56 (0.54 to 0.58)	0.60 (0.58 to 0.62)	0.60 (0.58 to 0.63)	0.62 (0.60 to 0.65)	0.52 (0.49 to 0.54)	0.56 (0.54 to 0.59)
60	0.80 (0.79 to 0.81)	0.82 (0.81 to 0.83)	0.82 (0.81 to 0.83)	0.83 (0.81 to 0.84)	0.77 (0.76 to 0.79)	0.80 (0.78 to 0.81)
65	ref	ref	ref	ref	ref	ref
70	1.13 (1.12 to 1.15)	1.12 (1.11 to 1.13)	1.12 (1.10 to 1.13)	1.12 (1.10 to 1.14)	1.16 (1.15 to 1.18)	1.15 (1.13 to 1.17)
75	1.20 (1.17 to 1.23)	1.18 (1.15 to 1.22)	1.17 (1.13 to 1.21)	1.19 (1.15 to 1.24)	1.25 (1.21 to 1.29)	1.23 (1.18 to 1.28)
80	1.22 (1.17 to 1.26)	1.21 (1.15 to 1.26)	1.19 (1.13 to 1.26)	1.24 (1.16 to 1.32)	1.28 (1.22 to 1.35)	1.26 (1.18 to 1.35)
85	1.22 (1.16 to 1.29)	1.22 (1.14 to 1.30)	1.20 (1.11 to 1.30)	1.27 (1.16 to 1.40)	1.29 (1.19 to 1.39)	1.28 (1.17 to 1.41)
90	1.22 (1.14 to 1.31)	1.23 (1.13 to 1.34)	1.21 (1.09 to 1.34)	1.31 (1.16 to 1.48)	1.29 (1.17 to 1.42)	1.30 (1.15 to 1.47)
95	1.22 (1.12 to 1.34)	1.24 (1.12 to 1.38)	1.22 (1.07 to 1.38)	1.35 (1.16 to 1.56)	1.30 (1.15 to 1.47)	1.31 (1.13 to 1.53)

Table 8. Estimated hazard ratios for age: from diagnosed with COVID-19 to hospitalised with COVID-19, stratified by calendar month

From diagnosed with COVID-19 to hospitalised with COVID-19				
Relative hazard ratios (95% CI) for age from models				
	March		April	
Age	Age only model	Age and comorbidity model	Age only model	Age and comorbidity model
20	0.064 (0.054 to 0.077)	0.08 (0.067 to 0.096)	0.13 (0.10 to 0.15)	0.15 (0.13 to 0.19)
25	0.085 (0.076 to 0.096)	0.10 (0.092 to 0.12)	0.16 (0.14 to 0.18)	0.19 (0.16 to 0.23)
30	0.11 (0.10 to 0.12)	0.13 (0.12 to 0.15)	0.20 (0.17 to 0.23)	0.24 (0.20 to 0.28)
35	0.14 (0.13 to 0.16)	0.17 (0.16 to 0.19)	0.23 (0.21 to 0.27)	0.28 (0.24 to 0.33)
40	0.17 (0.16 to 0.19)	0.21 (0.19 to 0.23)	0.28 (0.25 to 0.31)	0.34 (0.30 to 0.39)
45	0.22 (0.20 to 0.24)	0.27 (0.24 to 0.29)	0.34 (0.30 to 0.38)	0.41 (0.36 to 0.47)
50	0.33 (0.31 to 0.36)	0.38 (0.36 to 0.41)	0.45 (0.40 to 0.50)	0.53 (0.47 to 0.59)
55	0.54 (0.52 to 0.56)	0.59 (0.56 to 0.61)	0.62 (0.58 to 0.66)	0.68 (0.64 to 0.73)
60	0.79 (0.78 to 0.80)	0.81 (0.80 to 0.82)	0.82 (0.80 to 0.84)	0.85 (0.82 to 0.87)
65	1.00 (1.00 to 1.00)	1.00 (1.00 to 1.00)	1.00 (1.00 to 1.00)	1.00 (1.00 to 1.00)
70	1.15 (1.14 to 1.17)	1.13 (1.11 to 1.15)	1.15 (1.14 to 1.17)	1.13 (1.11 to 1.16)
75	1.24 (1.21 to 1.28)	1.21 (1.17 to 1.25)	1.28 (1.23 to 1.32)	1.24 (1.18 to 1.30)
80	1.30 (1.24 to 1.37)	1.26 (1.19 to 1.34)	1.37 (1.29 to 1.46)	1.33 (1.23 to 1.44)
85	1.36 (1.28 to 1.46)	1.32 (1.22 to 1.43)	1.45 (1.32 to 1.59)	1.41 (1.26 to 1.58)
90	1.42 (1.31 to 1.55)	1.37 (1.24 to 1.52)	1.53 (1.35 to 1.73)	1.49 (1.29 to 1.74)
95	1.49 (1.34 to 1.66)	1.43 (1.26 to 1.62)	1.62 (1.38 to 1.88)	1.58 (1.31 to 1.91)

Table 9. Estimated hazard ratios for age: from diagnosed with COVID-19 to hospitalised with COVID-19, stratified by region

From diagnosed with COVID-19 to hospitalised with COVID-19								
**Relative hazard ratios (95% CI) for age from models**								
	**Barcelona**		**Girona**		**Lleida**		**Tarragona**	
**Age**	**Age only model**	**Age and comorbidity model**	**Age only model**	**Age and comorbidity model**	**Age only model**	**Age and comorbidity model**	**Age only model**	**Age and comorbidity model**
20	0.07 (0.06 to 0.082)	0.085 (0.072 to 0.10)	0.13 (0.097 to 0.17)	0.17 (0.12 to 0.23)	0.052 (0.031 to 0.089)	0.063 (0.037 to 0.11)	0.084 (0.048 to 0.15)	0.092 (0.05 to 0.17)
25	0.097 (0.087 to 0.11)	0.12 (0.10 to 0.13)	0.15 (0.11 to 0.19)	0.18 (0.14 to 0.25)	0.072 (0.045 to 0.11)	0.086 (0.053 to 0.14)	0.084 (0.049 to 0.14)	0.091 (0.051 to 0.16)
30	0.13 (0.12 to 0.14)	0.15 (0.14 to 0.17)	0.16 (0.12 to 0.21)	0.21 (0.15 to 0.27)	0.095 (0.062 to 0.15)	0.11 (0.072 to 0.18)	0.088 (0.051 to 0.15)	0.095 (0.053 to 0.17)
35	0.17 (0.15 to 0.18)	0.20 (0.18 to 0.21)	0.18 (0.14 to 0.23)	0.23 (0.18 to 0.30)	0.12 (0.084 to 0.17)	0.15 (0.099 to 0.21)	0.10 (0.062 to 0.16)	0.11 (0.064 to 0.18)
40	0.20 (0.19 to 0.21)	0.24 (0.22 to 0.26)	0.20 (0.16 to 0.26)	0.26 (0.20 to 0.33)	0.15 (0.11 to 0.21)	0.19 (0.13 to 0.26)	0.12 (0.081 to 0.19)	0.14 (0.086 to 0.22)
45	0.24 (0.23 to 0.26)	0.29 (0.27 to 0.31)	0.26 (0.21 to 0.33)	0.33 (0.25 to 0.42)	0.21 (0.16 to 0.30)	0.26 (0.19 to 0.37)	0.18 (0.12 to 0.29)	0.20 (0.12 to 0.32)
50	0.35 (0.33 to 0.37)	0.40 (0.38 to 0.43)	0.39 (0.33 to 0.47)	0.46 (0.38 to 0.56)	0.35 (0.27 to 0.44)	0.40 (0.31 to 0.52)	0.32 (0.22 to 0.45)	0.34 (0.23 to 0.49)
55	0.55 (0.53 to 0.57)	0.59 (0.57 to 0.62)	0.60 (0.55 to 0.65)	0.65 (0.59 to 0.72)	0.56 (0.49 to 0.63)	0.61 (0.54 to 0.70)	0.54 (0.45 to 0.65)	0.56 (0.46 to 0.68)
60	0.79 (0.78 to 0.80)	0.81 (0.80 to 0.83)	0.82 (0.79 to 0.84)	0.84 (0.81 to 0.88)	0.80 (0.76 to 0.83)	0.83 (0.79 to 0.88)	0.80 (0.75 to 0.85)	0.80 (0.74 to 0.86)
65	1.00 (1.00 to 1.00)	1.00 (1.00 to 1.00)	1.00 (1.00 to 1.00)	1.00 (1.00 to 1.00)	1.00 (1.00 to 1.00)	1.00 (1.00 to 1.00)	1.00 (1.00 to 1.00)	1.00 (1.00 to 1.00)
70	1.14 (1.12 to 1.15)	1.12 (1.11 to 1.14)	1.14 (1.10 to 1.18)	1.11 (1.06 to 1.16)	1.14 (1.09 to 1.19)	1.10 (1.04 to 1.16)	1.12 (1.05 to 1.19)	1.13 (1.04 to 1.22)
75	1.20 (1.17 to 1.22)	1.19 (1.15 to 1.22)	1.23 (1.13 to 1.33)	1.19 (1.07 to 1.31)	1.22 (1.10 to 1.34)	1.13 (1.00 to 1.28)	1.15 (1.00 to 1.33)	1.19 (1.00 to 1.41)
80	1.21 (1.16 to 1.26)	1.21 (1.15 to 1.27)	1.29 (1.13 to 1.47)	1.24 (1.05 to 1.46)	1.25 (1.06 to 1.47)	1.13 (0.92 to 1.38)	1.14 (0.90 to 1.44)	1.20 (0.90 to 1.60)
85	1.20 (1.13 to 1.27)	1.22 (1.13 to 1.31)	1.35 (1.12 to 1.62)	1.28 (1.02 to 1.62)	1.26 (0.99 to 1.60)	1.11 (0.82 to 1.48)	1.12 (0.80 to 1.57)	1.21 (0.81 to 1.81)
90	1.20 (1.11 to 1.29)	1.22 (1.11 to 1.34)	1.42 (1.12 to 1.80)	1.34 (0.99 to 1.80)	1.27 (0.93 to 1.74)	1.08 (0.74 to 1.59)	1.10 (0.71 to 1.70)	1.22 (0.72 to 2.04)
95	1.19 (1.08 to 1.31)	1.23 (1.10 to 1.38)	1.48 (1.11 to 1.99)	1.39 (0.97 to 2.00)	1.29 (0.87 to 1.90)	1.06 (0.67 to 1.70)	1.08 (0.63 to 1.85)	1.22 (0.65 to 2.31)

Table 10. Estimated hazard ratios for age: from diagnosed with COVID-19 to death, overall and stratified by age

**From diagnosed with COVID-19 to death**						
**Relative hazard ratios (95% CI) for age from models**						
	**Overall**		**Male**		**Female**	
**Age**	**Age only model**	**Age and comorbidity model**	**Age only model**	**Age and comorbidity model**	**Age only model**	**Age and comorbidity model**
20	0.0074 (0.0023 to 0.024)	0.0083 (0.0024 to 0.03)	0.43 (0.37 to 0.50)	0.0078 (0.0018 to 0.035)	0.0048 (0.0038 to 0.0062)	0.0084 (0.00091 to 0.079)
25	0.0089 (0.0038 to 0.021)	0.0099 (0.004 to 0.025)	0.53 (0.46 to 0.61)	0.0093 (0.003 to 0.029)	0.0058 (0.0047 to 0.0073)	0.011 (0.0023 to 0.048)
30	0.01 (0.0044 to 0.022)	0.011 (0.0047 to 0.027)	0.46 (0.41 to 0.53)	0.011 (0.0036 to 0.031)	0.0067 (0.0055 to 0.0082)	0.012 (0.0031 to 0.048)
35	0.0097 (0.0044 to 0.021)	0.012 (0.005 to 0.027)	0.24 (0.21 to 0.27)	0.011 (0.0041 to 0.031)	0.007 (0.0059 to 0.0083)	0.012 (0.003 to 0.047)
40	0.0081 (0.0046 to 0.014)	0.011 (0.006 to 0.02)	0.066 (0.059 to 0.073)	0.012 (0.0057 to 0.024)	0.0064 (0.0055 to 0.0075)	0.0094 (0.0033 to 0.027)
45	0.009 (0.006 to 0.014)	0.013 (0.0088 to 0.02)	0.025 (0.022 to 0.027)	0.016 (0.0092 to 0.027)	0.0076 (0.0067 to 0.0087)	0.0092 (0.0046 to 0.018)
50	0.023 (0.016 to 0.033)	0.033 (0.023 to 0.047)	0.039 (0.036 to 0.042)	0.039 (0.025 to 0.062)	0.02 (0.018 to 0.022)	0.022 (0.012 to 0.04)
55	0.095 (0.077 to 0.12)	0.12 (0.099 to 0.15)	0.15 (0.14 to 0.16)	0.14 (0.11 to 0.18)	0.086 (0.08 to 0.093)	0.093 (0.065 to 0.13)
60	0.35 (0.33 to 0.38)	0.40 (0.37 to 0.43)	0.50 (0.48 to 0.51)	0.43 (0.39 to 0.47)	0.33 (0.32 to 0.35)	0.35 (0.31 to 0.40)
65	ref	ref	ref	ref	ref	ref
70	2.30 (2.21 to 2.39)	2.05 (1.96 to 2.13)	1.41 (1.34 to 1.47)	1.90 (1.81 to 2.00)	2.44 (2.33 to 2.56)	2.27 (2.11 to 2.44)
75	4.56 (4.30 to 4.82)	3.62 (3.41 to 3.85)	1.54 (1.40 to 1.69)	3.14 (2.90 to 3.39)	5.15 (4.67 to 5.67)	4.37 (3.93 to 4.87)
80	8.29 (7.76 to 8.85)	5.91 (5.48 to 6.36)	1.48 (1.28 to 1.71)	4.79 (4.31 to 5.34)	9.90 (8.52 to 11.51)	7.63 (6.75 to 8.62)
85	14.69 (13.58 to 15.89)	9.38 (8.54 to 10.29)	1.37 (1.12 to 1.67)	7.20 (6.24 to 8.30)	18.40 (15.00 to 22.58)	12.82 (11.16 to 14.73)
90	26.00 (23.62 to 28.62)	14.88 (13.25 to 16.70)	1.27 (0.99 to 1.63)	10.80 (8.99 to 12.96)	34.14 (26.35 to 44.23)	21.49 (18.25 to 25.31)
95	46.03 (40.97 to 51.71)	23.60 (20.52 to 27.16)	1.18 (0.87 to 1.59)	16.20 (12.94 to 20.28)	63.33 (46.30 to 86.64)	36.03 (29.71 to 43.69)

Table 11. Estimated hazard ratios for age: from diagnosed with COVID-19 to death, stratified by calendar month

**From diagnosed with COVID-19 to death**				
**Relative hazard ratios (95% CI) for age from models**				
	**March**		**April**	
**Age**	**Age only model**	**Age and comorbidity model**	**Age only model**	**Age and comorbidity model**
20	0.0093 (0.0019 to 0.045)	0.013 (0.0027 to 0.062)	0.0031 (0.0003 to 0.032)	0.0052 (0.00077 to 0.035)
25	0.011 (0.0039 to 0.033)	0.015 (0.0051 to 0.045)	0.0039 (0.00065 to 0.024)	0.0057 (0.0011 to 0.031)
30	0.013 (0.0052 to 0.034)	0.017 (0.0067 to 0.046)	0.0047 (0.00081 to 0.027)	0.0062 (0.0011 to 0.034)
35	0.014 (0.0053 to 0.035)	0.018 (0.007 to 0.048)	0.005 (0.00099 to 0.025)	0.0067 (0.0015 to 0.031)
40	0.012 (0.0058 to 0.025)	0.017 (0.008 to 0.035)	0.005 (0.0017 to 0.015)	0.0076 (0.0028 to 0.021)
45	0.013 (0.0073 to 0.022)	0.018 (0.01 to 0.032)	0.0073 (0.0039 to 0.014)	0.012 (0.0064 to 0.022)
50	0.029 (0.018 to 0.048)	0.041 (0.025 to 0.067)	0.021 (0.013 to 0.035)	0.033 (0.02 to 0.054)
55	0.11 (0.082 to 0.15)	0.14 (0.10 to 0.18)	0.091 (0.067 to 0.12)	0.12 (0.09 to 0.16)
60	0.37 (0.34 to 0.41)	0.42 (0.38 to 0.46)	0.35 (0.31 to 0.39)	0.40 (0.35 to 0.45)
65	1.00 (1.00 to 1.00)	1.00 (1.00 to 1.00)	1.00 (1.00 to 1.00)	1.00 (1.00 to 1.00)
70	2.25 (2.15 to 2.35)	2.01 (1.91 to 2.11)	2.29 (2.14 to 2.45)	2.03 (1.90 to 2.18)
75	4.51 (4.22 to 4.82)	3.59 (3.33 to 3.87)	4.43 (4.02 to 4.88)	3.53 (3.19 to 3.90)
80	8.61 (7.91 to 9.37)	6.12 (5.52 to 6.78)	7.63 (6.84 to 8.51)	5.50 (4.89 to 6.19)
85	16.34 (14.60 to 18.27)	10.37 (9.04 to 11.90)	12.41 (11.01 to 13.99)	8.13 (7.11 to 9.30)
90	31.00 (26.88 to 35.77)	17.57 (14.74 to 20.93)	20.00 (17.43 to 22.95)	11.92 (10.16 to 13.98)
95	58.84 (49.37 to 70.13)	29.76 (24.01 to 36.88)	32.23 (27.42 to 37.89)	17.46 (14.44 to 21.11)

Table 12. Estimated hazard ratios for age: from diagnosed with COVID-19 to death, stratified by region

From diagnosed with COVID-19 to death								
Relative hazard ratios (95% CI) for age from models								
	**Barcelona**		**Girona**		**Lleida**		**Tarragona**	
**Age**	**Age only model**	**Age and comorbidity model**	**Age only model**	**Age and comorbidity model**	**Age only model**	**Age and comorbidity model**	**Age only model**	**Age and comorbidity model**
20	0.0088 (0.0028 to 0.028)	0.011 (0.0033 to 0.035)	0.000044 (0.000023 to 0.000084)	0.0000000015 (0.00000000082 to 0.0000000029)	0.000026 (0.0000092 to 0.000075)	0.00077 (0.00000000000064 to 931,893.57)	0.000000017 (0.0000000067 to 0.000000041)	0.0000000000000000000087 (0.0000000000000000000035 to 0.000000000000000000022)
25	0.009 (0.0037 to 0.022)	0.01 (0.004 to 0.027)	0.000031 (0.000017 to 0.000055)	0.000007 (0.0000039 to 0.000012)	0.00051 (0.0002 to 0.0013)	0.00076 (0.00000000000023 to 2,497,758.62)	0.00003 (0.000013 to 0.000068)	0.0000000000059 (0.0000000000025 to 0.000000000014)
30	0.0091 (0.0037 to 0.022)	0.01 (0.004 to 0.027)	0.00011 (0.000064 to 0.00018)	0.0025 (0.0015 to 0.0042)	0.0037 (0.0016 to 0.0088)	0.0007 (0.00000000000037 to 1,332,760.23)	0.0062 (0.003 to 0.013)	0.000024 (0.000011 to 0.000051)
35	0.0089 (0.0037 to 0.021)	0.01 (0.0041 to 0.026)	0.0032 (0.002 to 0.0051)	0.032 (0.02 to 0.051)	0.0082 (0.0039 to 0.018)	0.00061 (0.000000000028 to 13,412.02)	0.052 (0.027 to 0.10)	0.048 (0.024 to 0.094)
40	0.0083 (0.0043 to 0.016)	0.011 (0.0054 to 0.021)	0.41 (0.27 to 0.63)	0.02 (0.014 to 0.03)	0.0076 (0.004 to 0.015)	0.00063 (0.000000038 to 10.37)	0.021 (0.012 to 0.037)	0.048 (0.026 to 0.086)
45	0.01 (0.0066 to 0.016)	0.015 (0.0094 to 0.023)	9.53 (6.67 to 13.61)	0.0099 (0.007 to 0.014)	0.009 (0.0051 to 0.016)	0.0014 (0.000011 to 0.17)	0.0072 (0.0044 to 0.012)	0.0075 (0.0045 to 0.012)
50	0.025 (0.017 to 0.036)	0.036 (0.025 to 0.052)	6.86 (5.13 to 9.17)	0.024 (0.018 to 0.031)	0.025 (0.016 to 0.039)	0.0077 (0.0003 to 0.20)	0.015 (0.01 to 0.023)	0.012 (0.0081 to 0.018)
55	0.097 (0.077 to 0.12)	0.13 (0.099 to 0.16)	1.55 (1.26 to 1.92)	0.11 (0.09 to 0.13)	0.10 (0.075 to 0.14)	0.057 (0.0093 to 0.35)	0.08 (0.06 to 0.11)	0.07 (0.052 to 0.095)
60	0.35 (0.32 to 0.39)	0.40 (0.37 to 0.44)	0.81 (0.72 to 0.90)	0.39 (0.35 to 0.43)	0.36 (0.30 to 0.43)	0.29 (0.15 to 0.59)	0.34 (0.29 to 0.40)	0.33 (0.28 to 0.38)
65	1.00 (1.00 to 1.00)	1.00 (1.00 to 1.00)	1.00 (1.00 to 1.00)	1.00 (1.00 to 1.00)	1.00 (1.00 to 1.00)	1.00 (1.00 to 1.00)	1.00 (1.00 to 1.00)	1.00 (1.00 to 1.00)
70	2.30 (2.20 to 2.40)	2.03 (1.94 to 2.13)	2.39 (2.10 to 2.71)	2.05 (1.82 to 2.31)	2.34 (1.93 to 2.84)	2.46 (1.72 to 3.52)	2.24 (1.88 to 2.68)	2.22 (1.85 to 2.66)

75	4.56 (4.29 to 4.86)	3.59 (3.36 to 3.84)	8.82 (6.78 to 11.48)	3.60 (2.82 to 4.61)	4.86 (3.26 to 7.25)	4.82 (2.92 to 7.97)	4.19 (2.91 to 6.03)	3.97 (2.73 to 5.76)
80	8.32 (7.75 to 8.94)	5.84 (5.38 to 6.34)	40.53 (27.04 to 60.75)	5.86 (4.02 to 8.55)	9.39 (5.08 to 17.36)	8.28 (4.78 to 14.34)	7.11 (4.06 to 12.46)	6.35 (3.58 to 11.27)
85	14.79 (13.57 to 16.11)	9.24 (8.35 to 10.24)	193.13 (111.81 to 333.61)	9.41 (5.65 to 15.66)	17.74 (7.72 to 40.79)	13.63 (7.46 to 24.90)	11.84 (5.54 to 25.30)	9.94 (4.58 to 21.60)
90	26.25 (23.65 to 29.15)	14.63 (12.89 to 16.61)	920.29 (462.28 to 1,832.08)	15.10 (7.95 to 28.67)	33.50 (11.71 to 95.79)	22.42 (11.25 to 44.69)	19.73 (7.58 to 51.36)	15.56 (5.85 to 41.38)
95	46.62 (41.07 to 52.92)	23.16 (19.86 to 27.01)	4,385.19 (1,911.29 to 10,061.20)	24.23 (11.18 to 52.52)	63.24 (17.78 to 224.96)	36.88 (16.58 to 82.07)	32.87 (10.36 to 104.27)	24.36 (7.48 to 79.30)

Table 13. Estimated hazard ratios for age: from hospitalised with COVID-19 to death, overall and stratified by age

From hospitalised with COVID-19 to death						
**Relative hazard ratios (95% CI) for age from models**						
	**Overall**		**Male**		**Female**	
Age	Age only model	Age and comorbidity model	Age only model	Age and comorbidity model	Age only model	Age and comorbidity model
20	0.052 (0.02 to 0.14)	0.072 (0.028 to 0.19)	0.054 (0.016 to 0.18)	0.072 (0.022 to 0.24)	0.057 (0.011 to 0.29)	0.076 (0.015 to 0.37)
25	0.066 (0.03 to 0.15)	0.088 (0.041 to 0.19)	0.067 (0.025 to 0.18)	0.088 (0.033 to 0.24)	0.074 (0.02 to 0.27)	0.093 (0.026 to 0.33)
30	0.084 (0.045 to 0.16)	0.11 (0.059 to 0.20)	0.084 (0.039 to 0.18)	0.11 (0.05 to 0.23)	0.095 (0.034 to 0.26)	0.11 (0.042 to 0.31)
35	0.11 (0.068 to 0.17)	0.13 (0.086 to 0.21)	0.10 (0.059 to 0.18)	0.13 (0.075 to 0.24)	0.12 (0.058 to 0.26)	0.14 (0.067 to 0.29)
40	0.14 (0.099 to 0.19)	0.17 (0.12 to 0.23)	0.13 (0.087 to 0.19)	0.16 (0.11 to 0.24)	0.16 (0.093 to 0.27)	0.17 (0.10 to 0.29)
45	0.18 (0.14 to 0.23)	0.21 (0.16 to 0.27)	0.17 (0.12 to 0.23)	0.20 (0.15 to 0.28)	0.21 (0.14 to 0.32)	0.22 (0.14 to 0.34)
50	0.25 (0.20 to 0.31)	0.28 (0.22 to 0.35)	0.23 (0.17 to 0.30)	0.27 (0.20 to 0.36)	0.28 (0.19 to 0.42)	0.29 (0.19 to 0.44)
55	0.37 (0.30 to 0.44)	0.40 (0.34 to 0.49)	0.34 (0.27 to 0.43)	0.39 (0.31 to 0.50)	0.40 (0.30 to 0.55)	0.41 (0.30 to 0.56)
60	0.59 (0.54 to 0.65)	0.63 (0.58 to 0.69)	0.58 (0.52 to 0.65)	0.63 (0.56 to 0.70)	0.61 (0.53 to 0.70)	0.63 (0.54 to 0.72)
65	ref	ref	ref	ref	ref	ref
70	1.69 (1.61 to 1.78)	1.54 (1.46 to 1.62)	1.63 (1.53 to 1.75)	1.50 (1.40 to 1.61)	1.81 (1.67 to 1.95)	1.64 (1.51 to 1.78)
75	2.75 (2.47 to 3.06)	2.25 (2.01 to 2.52)	2.51 (2.17 to 2.89)	2.12 (1.83 to 2.46)	3.30 (2.78 to 3.91)	2.62 (2.20 to 3.13)
80	4.16 (3.64 to 4.75)	3.15 (2.74 to 3.62)	3.63 (3.08 to 4.29)	2.91 (2.44 to 3.47)	5.44 (4.36 to 6.79)	3.91 (3.10 to 4.93)
85	5.93 (5.23 to 6.73)	4.25 (3.71 to 4.88)	5.10 (4.36 to 5.96)	3.93 (3.32 to 4.66)	8.12 (6.53 to 10.11)	5.44 (4.30 to 6.88)
90	8.28 (7.34 to 9.34)	5.68 (4.97 to 6.50)	7.07 (6.05 to 8.26)	5.28 (4.44 to 6.29)	11.53 (9.40 to 14.13)	7.33 (5.84 to 9.19)
95	11.53 (10.10 to 13.17)	7.58 (6.53 to 8.81)	9.81 (8.19 to 11.76)	7.10 (5.79 to 8.69)	16.22 (13.16 to 19.99)	9.81 (7.72 to 12.47)

Table 14. Estimated hazard ratios for age: from hospitalised with COVID-19 to death, stratified by calendar month

From hospitalised with COVID-19 to death				
**Relative hazard ratios (95% CI) for age from models**				
	**March**		**April**	
**Age**	**Age only model**	**Age and comorbidity model**	**Age only model**	**Age and comorbidity model**
20	0.052 (0.017 to 0.16)	0.071 (0.023 to 0.22)	0.041 (0.0062 to 0.27)	0.056 (0.0087 to 0.36)
25	0.068 (0.027 to 0.17)	0.089 (0.035 to 0.22)	0.054 (0.012 to 0.25)	0.071 (0.016 to 0.32)
30	0.087 (0.042 to 0.18)	0.11 (0.054 to 0.23)	0.072 (0.022 to 0.23)	0.091 (0.029 to 0.28)
35	0.11 (0.065 to 0.20)	0.14 (0.081 to 0.24)	0.095 (0.041 to 0.22)	0.12 (0.051 to 0.27)
40	0.15 (0.099 to 0.22)	0.18 (0.12 to 0.26)	0.13 (0.07 to 0.23)	0.15 (0.083 to 0.27)
45	0.19 (0.14 to 0.26)	0.23 (0.17 to 0.30)	0.17 (0.10 to 0.28)	0.20 (0.12 to 0.33)
50	0.26 (0.20 to 0.34)	0.30 (0.23 to 0.39)	0.25 (0.16 to 0.39)	0.28 (0.17 to 0.44)
55	0.38 (0.31 to 0.47)	0.42 (0.34 to 0.52)	0.37 (0.27 to 0.52)	0.41 (0.30 to 0.58)
60	0.60 (0.53 to 0.67)	0.63 (0.57 to 0.71)	0.60 (0.53 to 0.68)	0.64 (0.57 to 0.74)
65	1.00 (1.00 to 1.00)	1.00 (1.00 to 1.00)	1.00 (1.00 to 1.00)	1.00 (1.00 to 1.00)
70	1.75 (1.64 to 1.85)	1.59 (1.50 to 1.69)	1.68 (1.53 to 1.84)	1.51 (1.36 to 1.66)
75	2.92 (2.56 to 3.32)	2.41 (2.10 to 2.75)	2.77 (2.27 to 3.38)	2.22 (1.80 to 2.74)
80	4.25 (3.63 to 4.96)	3.26 (2.77 to 3.84)	4.39 (3.42 to 5.65)	3.25 (2.49 to 4.23)
85	5.60 (4.84 to 6.47)	4.09 (3.50 to 4.79)	6.73 (5.25 to 8.63)	4.73 (3.63 to 6.18)
90	7.15 (6.19 to 8.26)	5.01 (4.27 to 5.89)	10.13 (8.02 to 12.81)	6.88 (5.32 to 8.90)
95	9.12 (7.72 to 10.79)	6.14 (5.09 to 7.42)	15.21 (11.95 to 19.36)	10.01 (7.64 to 13.10)

Table 15. Estimated hazard ratios for age: from hospitalised with COVID-19 to death, stratified by region

From hospitalised with COVID-19 to death								
**Relative hazard ratios (95% CI) for age from models**								
	**Barcelona**		**Girona**		**Lleida**		**Tarragona**	
**Age**	**Age only model**	**Age and comorbidity model**	**Age only model**	**Age and comorbidity model**	**Age only model**	**Age and comorbidity model**	**Age only model**	**Age and comorbidity model**
20	0.049 (0.017 to 0.14)	0.069 (0.024 to 0.20)	0.029 (0.016 to 0.051)	0.035 (0.017 to 0.071)	0.043 (0.02 to 0.091)	0.081 (0.032 to 0.21)	0.08 (0.028 to 0.22)	0.20 (0.056 to 0.71)
25	0.062 (0.026 to 0.15)	0.083 (0.035 to 0.20)	0.042 (0.025 to 0.071)	0.051 (0.027 to 0.095)	0.061 (0.031 to 0.12)	0.11 (0.046 to 0.25)	0.11 (0.042 to 0.26)	0.24 (0.077 to 0.73)
30	0.078 (0.039 to 0.15)	0.10 (0.051 to 0.20)	0.063 (0.04 to 0.099)	0.074 (0.043 to 0.13)	0.087 (0.048 to 0.16)	0.14 (0.068 to 0.29)	0.14 (0.063 to 0.31)	0.28 (0.11 to 0.76)
35	0.097 (0.059 to 0.16)	0.12 (0.074 to 0.20)	0.093 (0.064 to 0.14)	0.11 (0.067 to 0.17)	0.12 (0.074 to 0.20)	0.19 (0.10 to 0.35)	0.19 (0.093 to 0.37)	0.34 (0.15 to 0.79)
40	0.12 (0.086 to 0.17)	0.15 (0.10 to 0.21)	0.14 (0.10 to 0.19)	0.16 (0.10 to 0.23)	0.17 (0.11 to 0.26)	0.25 (0.15 to 0.42)	0.25 (0.14 to 0.44)	0.41 (0.20 to 0.82)
45	0.16 (0.12 to 0.21)	0.18 (0.14 to 0.24)	0.21 (0.16 to 0.27)	0.23 (0.16 to 0.31)	0.25 (0.18 to 0.35)	0.33 (0.22 to 0.50)	0.33 (0.21 to 0.51)	0.49 (0.28 to 0.86)
50	0.22 (0.17 to 0.28)	0.25 (0.19 to 0.32)	0.31 (0.25 to 0.37)	0.33 (0.26 to 0.41)	0.35 (0.27 to 0.45)	0.43 (0.32 to 0.59)	0.43 (0.31 to 0.61)	0.58 (0.38 to 0.89)
55	0.33 (0.27 to 0.41)	0.37 (0.30 to 0.45)	0.45 (0.40 to 0.52)	0.47 (0.41 to 0.56)	0.50 (0.42 to 0.59)	0.57 (0.46 to 0.71)	0.57 (0.45 to 0.72)	0.70 (0.53 to 0.93)
60	0.57 (0.52 to 0.63)	0.60 (0.55 to 0.66)	0.67 (0.63 to 0.72)	0.69 (0.64 to 0.75)	0.71 (0.65 to 0.77)	0.76 (0.68 to 0.84)	0.76 (0.67 to 0.85)	0.84 (0.73 to 0.96)
65	1.00 (1.00 to 1.00)	1.00 (1.00 to 1.00)	1.00 (1.00 to 1.00)	1.00 (1.00 to 1.00)	1.00 (1.00 to 1.00)	1.00 (1.00 to 1.00)	1.00 (1.00 to 1.00)	1.00 (1.00 to 1.00)
70	1.69 (1.61 to 1.78)	1.53 (1.45 to 1.62)	1.48 (1.39 to 1.58)	1.45 (1.34 to 1.57)	1.42 (1.30 to 1.54)	1.32 (1.19 to 1.47)	1.32 (1.18 to 1.49)	1.20 (1.04 to 1.38)
75	2.71 (2.42 to 3.03)	2.21 (1.96 to 2.48)	2.20 (1.94 to 2.50)	2.11 (1.80 to 2.46)	2.01 (1.70 to 2.38)	1.75 (1.42 to 2.16)	1.75 (1.39 to 2.21)	1.43 (1.08 to 1.90)
80	4.08 (3.54 to 4.69)	3.07 (2.65 to 3.56)	3.27 (2.70 to 3.96)	3.06 (2.42 to 3.87)	2.85 (2.22 to 3.66)	2.31 (1.69 to 3.17)	2.32 (1.65 to 3.28)	1.71 (1.12 to 2.61)
85	5.89 (5.15 to 6.73)	4.19 (3.63 to 4.84)	4.86 (3.76 to 6.27)	4.44 (3.25 to 6.07)	4.05 (2.90 to 5.65)	3.06 (2.01 to 4.65)	3.08 (1.94 to 4.87)	2.05 (1.17 to 3.60)
90	8.37 (7.37 to 9.51)	5.69 (4.94 to 6.57)	7.21 (5.24 to 9.93)	6.44 (4.36 to 9.53)	5.74 (3.78 to 8.71)	4.04 (2.40 to 6.83)	4.07 (2.30 to 7.23)	2.45 (1.21 to 4.95)
95	11.88 (10.33 to 13.67)	7.73 (6.59 to 9.07)	10.70 (7.29 to 15.71)	9.35 (5.85 to 14.96)	8.14 (4.93 to 13.42)	5.35 (2.85 to 10.02)	5.39 (2.71 to 10.73)	2.93 (1.26 to 6.82)

Table 16. Estimated hazard ratios for male sex: overall and stratified by age

	Overall			Age: 70 or younger			Over 70		
**Transition**	**unadjusted**	**adjusted age**	**adjusted age and comorbidity**	**unadjusted**	**adjusted age**	**adjusted age and comorbidity**	**unadjusted**	**adjusted age**	**adjusted age and comorbidity**
From general population to diagnosed with COVID-19	0.75 (0.74 to 0.76)	0.75 (0.74 to 0.75)	0.74 (0.73 to 0.75)	0.71 (0.70 to 0.72)	0.72 (0.71 to 0.73)	0.73 (0.72 to 0.74)	1.05 (1.01 to 1.09)	1.11 (1.07 to 1.15)	1.06 (1.02 to 1.11)
From general population to hospitalised with COVID-19	1.37 (1.31 to 1.43)	1.64 (1.57 to 1.72)	1.55 (1.48 to 1.62)	1.37 (1.28 to 1.45)	1.44 (1.35 to 1.53)	1.38 (1.30 to 1.48)	1.79 (1.68 to 1.90)	1.86 (1.75 to 1.98)	1.72 (1.61 to 1.84)
From diagnosed with COVID-19 to hospitalised with COVID-19	1.73 (1.66 to 1.80)	1.77 (1.70 to 1.85)	1.78 (1.70 to 1.86)	1.80 (1.71 to 1.89)	1.85 (1.76 to 1.95)	1.85 (1.76 to 1.95)	1.58 (1.46 to 1.70)	1.53 (1.42 to 1.65)	1.51 (1.39 to 1.64)
From diagnosed with COVID-19 to death	1.09 (1.01 to 1.18)	1.33 (1.23 to 1.44)	1.32 (1.22 to 1.44)	2.95 (2.23 to 3.90)	3.04 (2.30 to 4.02)	2.47 (1.87 to 3.26)	1.00 (0.92 to 1.09)	1.25 (1.15 to 1.36)	1.25 (1.15 to 1.37)
From hospitalised with COVID-19 to death	1.16 (1.07 to 1.25)	1.31 (1.21 to 1.42)	1.27 (1.17 to 1.38)	1.64 (1.36 to 1.99)	1.61 (1.33 to 1.95)	1.47 (1.21 to 1.79)	1.13 (1.04 to 1.24)	1.25 (1.15 to 1.37)	1.23 (1.13 to 1.35)

Table 17. Estimated hazard ratios for male sex: stratified by calendar month

	**March**			**April**		
**Transition**	**unadjusted**	**adjusted age**	**adjusted age and comorbidity**	**unadjusted**	**adjusted age**	**adjusted age and comorbidity**
From general population to diagnosed with COVID-19	0.80 (0.79 to 0.81)	0.79 (0.78 to 0.81)	0.79 (0.78 to 0.81)	0.68 (0.66 to 0.69)	0.68 (0.67 to 0.70)	0.68 (0.66 to 0.69)
From general population to hospitalised with COVID-19	1.47 (1.39 to 1.55)	1.74 (1.65 to 1.84)	1.65 (1.56 to 1.75)	1.20 (1.11 to 1.29)	1.45 (1.34 to 1.57)	1.36 (1.26 to 1.48)
From diagnosed with COVID-19 to hospitalised with COVID-19	1.79 (1.70 to 1.87)	1.79 (1.70 to 1.88)	1.79 (1.70 to 1.88)	1.45 (1.34 to 1.58)	1.55 (1.43 to 1.68)	1.53 (1.41 to 1.67)
From diagnosed with COVID-19 to death	1.31 (1.17 to 1.48)	1.45 (1.29 to 1.63)	1.47 (1.30 to 1.67)	0.98 (0.88 to 1.09)	1.28 (1.15 to 1.43)	1.23 (1.10 to 1.38)
From hospitalised with COVID-19 to death	1.34 (1.22 to 1.47)	1.49 (1.36 to 1.65)	1.42 (1.29 to 1.58)	0.91 (0.79 to 1.04)	1.04 (0.91 to 1.20)	1.04 (0.90 to 1.20)

Table 18. Estimated hazard ratios for male sex: stratified by region

	Barcelona			Girona			Lleida			Tarragona		
**Transition**	**unadjusted**	**adjusted age**	**adjusted age and comorbidity**	**unadjusted**	**adjusted age**	**adjusted age and comorbidity**	**unadjusted**	**adjusted age**	**adjusted age and comorbidity**	**unadjusted**	**adjusted age**	**adjusted age and comorbidity**
From general population to diagnosed with COVID-19	0.74 (0.73 to 0.75)	0.74 (0.73 to 0.75)	0.74 (0.73 to 0.75)	0.77 (0.74 to 0.81)	0.77 (0.74 to 0.80)	0.77 (0.74 to 0.80)	0.81 (0.77 to 0.85)	0.79 (0.75 to 0.84)	0.79 (0.75 to 0.83)	0.72 (0.68 to 0.77)	0.72 (0.67 to 0.76)	0.70 (0.65 to 0.74)
From general population to hospitalised with COVID-19	1.37 (1.30 to 1.43)	1.64 (1.56 to 1.72)	1.55 (1.48 to 1.63)	1.44 (1.19 to 1.75)	1.71 (1.41 to 2.08)	1.65 (1.35 to 2.01)	1.75 (1.32 to 2.32)	2.14 (1.61 to 2.85)	2.04 (1.52 to 2.73)	1.60 (1.20 to 2.14)	1.92 (1.44 to 2.57)	1.74 (1.29 to 2.34)
From diagnosed with COVID-19 to hospitalised with COVID-19	1.73 (1.66 to 1.81)	1.77 (1.69 to 1.85)	1.78 (1.70 to 1.86)	1.44 (1.24 to 1.68)	1.52 (1.31 to 1.78)	1.52 (1.30 to 1.78)	2.18 (1.78 to 2.68)	2.35 (1.91 to 2.89)	2.28 (1.85 to 2.81)	1.98 (1.50 to 2.61)	1.98 (1.50 to 2.61)	2.00 (1.51 to 2.64)
From diagnosed with COVID-19 to death	1.10 (1.01 to 1.20)	1.36 (1.25 to 1.48)	1.36 (1.24 to 1.49)	0.99 (0.76 to 1.30)	1.26 (0.96 to 1.66)	1.30 (0.97 to 1.74)	0.93 (0.64 to 1.36)	1.03 (0.70 to 1.52)	1.00 (0.65 to 1.54)	1.32 (0.88 to 1.99)	1.37 (0.90 to 2.07)	1.25 (0.81 to 1.93)
From hospitalised with COVID-19 to death	1.17 (1.08 to 1.27)	1.34 (1.23 to 1.46)	1.31 (1.20 to 1.43)	1.25 (0.91 to 1.72)	1.33 (0.96 to 1.83)	1.35 (0.96 to 1.89)	0.86 (0.57 to 1.31)	0.91 (0.60 to 1.39)	0.91 (0.58 to 1.42)	1.01 (0.55 to 1.84)	1.01 (0.55 to 1.85)	

Table 19. Estimated hazard ratios for comorbidities: overall and stratified by age

		Overall		Male- 70 or younger		Female- 70 or younger		Male- Over 70		Female- Over 70	
**Transition**	**Variable**	**Unadjusted**	**Adjusted for age and sex**	**Unadjusted**	**Adjusted for age**	**Unadjusted**	**Adjusted for age**	**Unadjusted**	**Adjusted for age**	**Unadjusted**	**Adjusted for age**
From general population to diagnosed with COVID-19	Charlson - 1:0	1.28 (1.26 to 1.31)	1.28 (1.25 to 1.30)	1.30 (1.25 to 1.35)	1.06 (1.03 to 1.11)	1.40 (1.36 to 1.45)	1.19 (1.15 to 1.23)	1.45 (1.32 to 1.59)	1.40 (1.27 to 1.53)	1.86 (1.73 to 2.01)	1.74 (1.62 to 1.88)
From general population to diagnosed with COVID-19	Charlson - 2:0	1.09 (1.07 to 1.12)	1.13 (1.10 to 1.16)	1.23 (1.17 to 1.29)	0.91 (0.87 to 0.96)	1.20 (1.15 to 1.25)	0.91 (0.88 to 0.95)	1.44 (1.33 to 1.57)	1.35 (1.24 to 1.47)	1.46 (1.36 to 1.56)	1.34 (1.26 to 1.44)
From general population to diagnosed with COVID-19	Charlson - 3+:0	1.27 (1.23 to 1.30)	1.48 (1.44 to 1.53)	1.20 (1.13 to 1.28)	0.87 (0.82 to 0.92)	1.26 (1.17 to 1.34)	0.93 (0.86 to 0.99)	1.96 (1.82 to 2.11)	1.75 (1.62 to 1.89)	2.28 (2.14 to 2.43)	1.95 (1.83 to 2.08)
From general population to diagnosed with COVID-19	Autoimmune condition	1.31 (1.28 to 1.34)	1.17 (1.14 to 1.20)	1.35 (1.29 to 1.41)	1.14 (1.09 to 1.20)	1.33 (1.28 to 1.37)	1.17 (1.13 to 1.21)	1.13 (1.03 to 1.24)	1.11 (1.01 to 1.23)	1.18 (1.10 to 1.27)	1.17 (1.09 to 1.26)
From general population to diagnosed with COVID-19	Chronic kidney disease	1.08 (1.05 to 1.12)	1.18 (1.14 to 1.22)	1.17 (1.07 to 1.28)	0.85 (0.77 to 0.93)	1.02 (0.92 to 1.12)	0.75 (0.68 to 0.82)	1.30 (1.22 to 1.39)	1.16 (1.08 to 1.23)	1.35 (1.28 to 1.42)	1.13 (1.07 to 1.20)
From general population to diagnosed with COVID-19	COPD	1.09 (1.05 to 1.14)	1.20 (1.15 to 1.25)	1.07 (0.99 to 1.15)	0.77 (0.71 to 0.83)	1.18 (1.08 to 1.29)	0.87 (0.80 to 0.95)	1.30 (1.21 to 1.40)	1.28 (1.19 to 1.38)	1.29 (1.15 to 1.44)	1.27 (1.13 to 1.42)
From general population to diagnosed with COVID-19	Dementia	2.62 (2.51 to 2.74)	3.09 (2.94 to 3.24)	NA	NA	NA	NA	8.32 (7.81 to 8.87)	2.90 (2.66 to 3.16)	3.59 (3.37 to 3.82)	3.08 (2.89 to 3.29)
From general population to diagnosed with COVID-19	Heart disease	1.13 (1.11 to 1.15)	1.23 (1.20 to 1.26)	1.24 (1.19 to 1.28)	0.98 (0.94 to 1.01)	1.32 (1.27 to 1.37)	1.05 (1.00 to 1.09)	1.25 (1.18 to 1.32)	1.15 (1.09 to 1.22)	1.34 (1.27 to 1.40)	1.19 (1.14 to 1.26)
From general population to diagnosed with COVID-19	Hyperlipidemia	1.18 (1.16 to 1.21)	1.11 (1.09 to 1.13)	1.37 (1.32 to 1.41)	1.10 (1.06 to 1.13)	1.24 (1.20 to 1.27)	0.94 (0.91 to 0.97)	1.00 (0.93 to 1.07)	1.06 (0.98 to 1.14)	0.91 (0.86 to 0.97)	0.97 (0.91 to 1.03)
From general population to diagnosed with COVID-19	Hypertension	1.11 (1.10 to 1.14)	1.10 (1.08 to 1.12)	1.31 (1.27 to 1.35)	0.97 (0.94 to 1.00)	1.23 (1.19 to 1.26)	0.91 (0.89 to 0.94)	0.95 (0.89 to 1.00)	0.97 (0.92 to 1.03)	1.02 (0.97 to 1.08)	1.02 (0.97 to 1.08)

From general population to diagnosed with COVID-19	Malignant neoplasm	1.06 (1.03 to 1.09)	1.06 (1.03 to 1.09)	1.15 (1.08 to 1.22)	0.85 (0.80 to 0.90)	1.13 (1.08 to 1.18)	0.87 (0.83 to 0.91)	1.12 (1.06 to 1.20)	1.08 (1.01 to 1.15)	1.08 (1.01 to 1.15)	1.05 (0.98 to 1.12)
From general population to diagnosed with COVID-19	Obesity	1.29 (1.27 to 1.31)	1.23 (1.21 to 1.25)	1.36 (1.32 to 1.39)	1.15 (1.11 to 1.18)	1.41 (1.38 to 1.44)	1.22 (1.19 to 1.25)	0.99 (0.93 to 1.05)	1.03 (0.97 to 1.09)	1.05 (0.99 to 1.10)	1.10 (1.04 to 1.15)
From general population to diagnosed with COVID-19	Type 2 diabetes	1.04 (1.02 to 1.07)	1.08 (1.05 to 1.11)	1.18 (1.13 to 1.24)	0.85 (0.82 to 0.90)	1.17 (1.11 to 1.23)	0.86 (0.82 to 0.90)	1.09 (1.02 to 1.16)	1.12 (1.05 to 1.19)	1.08 (1.01 to 1.15)	1.08 (1.01 to 1.15)
From general population to hospitalised with COVID-19	Charlson - 1:0	3.27 (3.06 to 3.51)	1.45 (1.35 to 1.56)	2.59 (2.28 to 2.94)	1.28 (1.12 to 1.45)	2.18 (1.85 to 2.57)	1.35 (1.15 to 1.60)	1.53 (1.33 to 1.76)	1.50 (1.30 to 1.73)	1.58 (1.36 to 1.83)	1.51 (1.30 to 1.76)
From general population to hospitalised with COVID-19	Charlson - 2:0	4.97 (4.67 to 5.29)	1.63 (1.53 to 1.74)	4.32 (3.79 to 4.91)	1.57 (1.37 to 1.79)	3.97 (3.44 to 4.57)	1.84 (1.59 to 2.13)	1.54 (1.36 to 1.75)	1.49 (1.31 to 1.69)	1.61 (1.41 to 1.83)	1.53 (1.34 to 1.75)
From general population to hospitalised with COVID-19	Charlson - 3+:0	9.25 (8.75 to 9.77)	2.33 (2.18 to 2.48)	6.27 (5.49 to 7.16)	2.08 (1.81 to 2.39)	7.12 (5.92 to 8.56)	2.98 (2.46 to 3.60)	2.25 (2.02 to 2.51)	2.11 (1.89 to 2.36)	2.55 (2.27 to 2.87)	2.32 (2.05 to 2.62)
From general population to hospitalised with COVID-19	Autoimmune condition	2.17 (2.02 to 2.34)	1.33 (1.24 to 1.43)	2.11 (1.81 to 2.46)	1.34 (1.15 to 1.57)	2.27 (1.93 to 2.66)	1.56 (1.33 to 1.83)	1.26 (1.10 to 1.45)	1.26 (1.10 to 1.44)	1.28 (1.11 to 1.47)	1.27 (1.10 to 1.45)
From general population to hospitalised with COVID-19	Chronic kidney disease	5.70 (5.38 to 6.04)	1.51 (1.41 to 1.60)	5.99 (5.06 to 7.09)	2.03 (1.71 to 2.41)	6.01 (4.78 to 7.56)	2.39 (1.90 to 3.02)	1.44 (1.31 to 1.57)	1.33 (1.21 to 1.46)	1.64 (1.48 to 1.81)	1.46 (1.31 to 1.62)
From general population to hospitalised with COVID-19	COPD	4.69 (4.35 to 5.06)	1.50 (1.39 to 1.63)	4.03 (3.41 to 4.77)	1.36 (1.14 to 1.61)	3.49 (2.61 to 4.67)	1.57 (1.18 to 2.09)	1.46 (1.32 to 1.62)	1.44 (1.30 to 1.60)	1.56 (1.28 to 1.90)	1.54 (1.26 to 1.88)
From general population to hospitalised with COVID-19	Dementia	7.53 (6.83 to 8.30)	1.86 (1.68 to 2.06)	NA	NA	NA	NA	1.94 (1.66 to 2.26)	1.74 (1.49 to 2.03)	2.32 (2.02 to 2.66)	2.02 (1.76 to 2.33)
From general population to hospitalised with COVID-19	Heart disease	4.58 (4.37 to 4.80)	1.43 (1.36 to 1.51)	3.30 (2.97 to 3.66)	1.37 (1.23 to 1.53)	3.09 (2.65 to 3.59)	1.58 (1.35 to 1.85)	1.42 (1.31 to 1.54)	1.35 (1.24 to 1.46)	1.53 (1.40 to 1.68)	1.42 (1.29 to 1.56)
From general population to hospitalised with COVID-19	Hyperlipidemia	2.26 (2.14 to 2.39)	1.17 (1.10 to 1.24)	2.79 (2.52 to 3.09)	1.34 (1.21 to 1.48)	2.68 (2.36 to 3.04)	1.24 (1.09 to 1.41)	1.10 (0.99 to 1.22)	1.15 (1.03 to 1.27)	0.90 (0.80 to 1.02)	0.95 (0.84 to 1.07)

From general population to hospitalised with COVID-19	Hypertension	3.04 (2.90 to 3.19)	1.11 (1.06 to 1.17)	3.22 (2.94 to 3.53)	1.16 (1.06 to 1.28)	3.43 (3.06 to 3.85)	1.50 (1.33 to 1.69)	0.96 (0.88 to 1.04)	0.98 (0.90 to 1.06)	0.98 (0.89 to 1.08)	0.98 (0.89 to 1.08)
From general population to hospitalised with COVID-19	Malignant neoplasm	3.64 (3.43 to 3.86)	1.24 (1.17 to 1.32)	3.70 (3.22 to 4.26)	1.33 (1.16 to 1.54)	3.05 (2.60 to 3.58)	1.46 (1.24 to 1.72)	1.15 (1.05 to 1.26)	1.11 (1.01 to 1.22)	1.20 (1.07 to 1.36)	1.18 (1.04 to 1.33)
From general population to hospitalised with COVID-19	Obesity	3.36 (3.21 to 3.51)	1.78 (1.70 to 1.87)	3.61 (3.31 to 3.93)	1.88 (1.72 to 2.06)	4.21 (3.82 to 4.64)	2.69 (2.43 to 2.97)	1.31 (1.20 to 1.42)	1.35 (1.24 to 1.47)	1.55 (1.41 to 1.70)	1.61 (1.47 to 1.77)
From general population to hospitalised with COVID-19	Type 2 diabetes	4.29 (4.06 to 4.53)	1.54 (1.46 to 1.63)	4.47 (4.02 to 4.98)	1.62 (1.45 to 1.81)	5.55 (4.82 to 6.39)	2.41 (2.09 to 2.79)	1.28 (1.16 to 1.40)	1.30 (1.19 to 1.42)	1.38 (1.23 to 1.55)	1.38 (1.24 to 1.55)
From diagnosed with COVID-19 to hospitalised with COVID-19	Charlson - 1:0	1.78 (1.66 to 1.90)	1.03 (0.96 to 1.11)	1.54 (1.38 to 1.72)	0.99 (0.89 to 1.11)	1.43 (1.25 to 1.62)	1.10 (0.97 to 1.26)	0.94 (0.79 to 1.12)	0.99 (0.83 to 1.18)	0.86 (0.72 to 1.01)	0.95 (0.80 to 1.13)
From diagnosed with COVID-19 to hospitalised with COVID-19	Charlson - 2:0	2.72 (2.56 to 2.90)	1.19 (1.11 to 1.27)	2.45 (2.18 to 2.76)	1.15 (1.01 to 1.30)	1.86 (1.62 to 2.13)	1.13 (0.98 to 1.30)	1.13 (0.97 to 1.32)	1.17 (1.01 to 1.37)	0.99 (0.85 to 1.15)	1.07 (0.92 to 1.25)
From diagnosed with COVID-19 to hospitalised with COVID-19	Charlson - 3+:0	3.68 (3.46 to 3.91)	1.20 (1.12 to 1.30)	2.52 (2.18 to 2.92)	1.07 (0.92 to 1.25)	3.23 (2.69 to 3.88)	1.71 (1.41 to 2.06)	1.01 (0.88 to 1.16)	1.10 (0.96 to 1.27)	0.98 (0.85 to 1.12)	1.17 (1.01 to 1.36)
From diagnosed with COVID-19 to hospitalised with COVID-19	Autoimmune condition	1.40 (1.30 to 1.51)	1.07 (1.00 to 1.16)	1.51 (1.32 to 1.72)	1.19 (1.05 to 1.36)	1.23 (1.06 to 1.42)	1.00 (0.87 to 1.16)	0.91 (0.76 to 1.10)	0.90 (0.75 to 1.09)	1.12 (0.95 to 1.31)	1.10 (0.93 to 1.29)
From diagnosed with COVID-19 to hospitalised with COVID-19	Chronic kidney disease	3.18 (2.97 to 3.41)	1.18 (1.09 to 1.27)	3.01 (2.49 to 3.63)	1.30 (1.08 to 1.57)	3.52 (2.77 to 4.48)	1.87 (1.47 to 2.39)	1.05 (0.93 to 1.18)	1.11 (0.98 to 1.25)	0.96 (0.84 to 1.08)	1.09 (0.96 to 1.24)
From diagnosed with COVID-19 to hospitalised with COVID-19	COPD	2.62 (2.39 to 2.88)	0.98 (0.89 to 1.08)	2.04 (1.69 to 2.47)	0.90 (0.75 to 1.09)	1.42 (1.02 to 1.99)	0.76 (0.54 to 1.07)	1.00 (0.87 to 1.14)	1.01 (0.88 to 1.15)	1.14 (0.89 to 1.46)	1.14 (0.89 to 1.45)
From diagnosed with COVID-19 to hospitalised with COVID-19	Dementia	1.98 (1.77 to 2.23)	0.66 (0.58 to 0.75)	NA	NA	NA	NA	0.53 (0.44 to 0.65)	0.57 (0.46 to 0.69)	0.66 (0.56 to 0.78)	0.77 (0.65 to 0.91)
From diagnosed with COVID-19 to hospitalised with COVID-19	Heart disease	2.51 (2.39 to 2.64)	1.07 (1.01 to 1.13)	1.80 (1.62 to 1.99)	0.98 (0.88 to 1.09)	1.64 (1.43 to 1.89)	1.06 (0.92 to 1.23)	1.03 (0.93 to 1.15)	1.08 (0.97 to 1.20)	1.02 (0.91 to 1.14)	1.09 (0.97 to 1.22)

From diagnosed with COVID-19 to hospitalised with COVID-19	Hyperlipidemia	1.79 (1.69 to 1.89)	1.10 (1.04 to 1.16)	1.74 (1.59 to 1.90)	1.12 (1.02 to 1.22)	1.90 (1.71 to 2.11)	1.15 (1.04 to 1.29)	1.11 (0.97 to 1.27)	1.07 (0.93 to 1.22)	1.05 (0.91 to 1.21)	1.00 (0.87 to 1.15)
From diagnosed with COVID-19 to hospitalised with COVID-19	Hypertension	2.24 (2.14 to 2.36)	1.08 (1.02 to 1.13)	2.14 (1.97 to 2.31)	1.07 (0.99 to 1.17)	2.18 (1.98 to 2.41)	1.30 (1.18 to 1.44)	0.93 (0.84 to 1.04)	0.92 (0.82 to 1.02)	0.98 (0.87 to 1.10)	0.99 (0.88 to 1.11)
From diagnosed with COVID-19 to hospitalised with COVID-19	Malignant neoplasm	2.32 (2.18 to 2.48)	1.05 (0.98 to 1.13)	2.04 (1.77 to 2.36)	0.94 (0.81 to 1.09)	1.85 (1.59 to 2.15)	1.16 (1.00 to 1.35)	1.00 (0.89 to 1.12)	1.01 (0.90 to 1.14)	1.06 (0.91 to 1.23)	1.07 (0.92 to 1.23)
From diagnosed with COVID-19 to hospitalised with COVID-19	Obesity	2.21 (2.12 to 2.31)	1.57 (1.50 to 1.64)	2.20 (2.05 to 2.37)	1.43 (1.33 to 1.55)	2.53 (2.35 to 2.74)	2.11 (1.95 to 2.28)	1.19 (1.07 to 1.33)	1.15 (1.03 to 1.29)	1.36 (1.22 to 1.52)	1.30 (1.16 to 1.45)
From diagnosed with COVID-19 to hospitalised with COVID-19	Type 2 diabetes	3.02 (2.85 to 3.20)	1.34 (1.26 to 1.42)	2.72 (2.46 to 3.01)	1.31 (1.18 to 1.46)	3.23 (2.83 to 3.68)	1.88 (1.64 to 2.15)	1.10 (0.98 to 1.24)	1.09 (0.97 to 1.23)	1.21 (1.06 to 1.39)	1.21 (1.05 to 1.38)
From diagnosed with COVID-19 to death	Charlson - 1:0	13.80 (12.07 to 15.78)	2.45 (2.13 to 2.81)	4.04 (2.48 to 6.59)	1.79 (1.09 to 2.94)	8.72 (4.23 to 17.95)	5.42 (2.62 to 11.22)	2.14 (1.68 to 2.71)	1.75 (1.38 to 2.23)	3.76 (3.12 to 4.53)	2.58 (2.14 to 3.11)
From diagnosed with COVID-19 to death	Charlson - 2:0	17.11 (14.92 to 19.63)	1.68 (1.46 to 1.94)	10.27 (6.55 to 16.10)	2.65 (1.66 to 4.22)	15.09 (7.53 to 30.22)	6.12 (3.01 to 12.44)	1.43 (1.12 to 1.81)	1.26 (0.99 to 1.60)	2.24 (1.85 to 2.72)	1.56 (1.28 to 1.89)
From diagnosed with COVID-19 to death	Charlson - 3+:0	64.94 (57.77 to 73.00)	2.59 (2.28 to 2.94)	26.63 (17.92 to 39.56)	5.50 (3.60 to 8.41)	98.99 (54.16 to 180.92)	31.22 (16.55 to 58.89)	2.90 (2.38 to 3.54)	2.03 (1.66 to 2.48)	4.45 (3.75 to 5.27)	2.38 (2.00 to 2.83)
From diagnosed with COVID-19 to death	Autoimmune condition	1.90 (1.68 to 2.16)	1.12 (0.99 to 1.27)	3.29 (2.08 to 5.22)	2.32 (1.47 to 3.69)	2.20 (1.10 to 4.42)	1.43 (0.71 to 2.87)	1.01 (0.81 to 1.25)	1.10 (0.89 to 1.37)	0.93 (0.79 to 1.10)	1.05 (0.89 to 1.24)
From diagnosed with COVID-19 to death	Chronic kidney disease	18.46 (17.01 to 20.03)	1.24 (1.14 to 1.35)	13.21 (8.08 to 21.62)	2.65 (1.60 to 4.39)	35.79 (19.97 to 64.13)	9.62 (5.26 to 17.60)	1.70 (1.49 to 1.94)	1.33 (1.16 to 1.52)	1.78 (1.60 to 1.99)	1.12 (1.00 to 1.25)
From diagnosed with COVID-19 to death	COPD	6.01 (5.29 to 6.83)	1.07 (0.94 to 1.22)	6.53 (3.77 to 11.30)	1.43 (0.82 to 2.50)	14.66 (7.03 to 30.56)	4.90 (2.34 to 10.27)	1.09 (0.93 to 1.28)	1.07 (0.91 to 1.26)	0.98 (0.76 to 1.27)	1.02 (0.79 to 1.31)
From diagnosed with COVID-19 to death	Dementia	53.84 (49.64 to 58.38)	2.84 (2.60 to 3.09)	NA	NA	NA	NA	4.23 (3.70 to 4.85)	2.78 (2.41 to 3.20)	4.93 (4.43 to 5.49)	2.80 (2.51 to 3.13)
From diagnosed with COVID-19 to death	Heart disease	8.47 (7.83 to 9.15)	0.96 (0.88 to 1.03)	5.82 (4.13 to 8.20)	1.83 (1.28 to 2.60)	5.50 (3.16 to 9.58)	2.08 (1.18 to 3.66)	1.19 (1.05 to 1.35)	0.95 (0.84 to 1.08)	1.14 (1.03 to 1.27)	0.93 (0.84 to 1.04)
From diagnosed with COVID-19 to death	Hyperlipidemia	1.56 (1.40 to 1.74)	1.01 (0.91 to 1.13)	1.52 (0.98 to 2.37)	0.75 (0.48 to 1.17)	3.56 (2.09 to 6.06)	1.28 (0.75 to 2.20)	0.73 (0.61 to 0.88)	1.00 (0.83 to 1.21)	0.81 (0.70 to 0.94)	1.02 (0.88 to 1.18)
From diagnosed with COVID-19 to death	Hypertension	3.81 (3.52 to 4.14)	1.01 (0.93 to 1.10)	2.77 (1.94 to 3.97)	0.79 (0.55 to 1.14)	4.35 (2.64 to 7.17)	1.51 (0.91 to 2.51)	0.94 (0.82 to 1.07)	1.01 (0.88 to 1.15)	1.05 (0.94 to 1.17)	1.00 (0.90 to 1.12)

From diagnosed with COVID-19 to death	Malignant neoplasm	5.89 (5.37 to 6.45)	1.13 (1.03 to 1.24)	8.34 (5.56 to 12.51)	1.91 (1.26 to 2.90)	16.70 (10.45 to 26.68)	6.89 (4.27 to 11.11)	1.10 (0.95 to 1.26)	1.03 (0.90 to 1.19)	1.14 (0.99 to 1.31)	1.10 (0.96 to 1.26)
From diagnosed with COVID-19 to death	Obesity	1.58 (1.45 to 1.73)	0.90 (0.83 to 0.99)	2.53 (1.79 to 3.57)	1.12 (0.79 to 1.59)	2.83 (1.77 to 4.52)	1.82 (1.14 to 2.92)	0.69 (0.59 to 0.80)	0.88 (0.76 to 1.03)	0.63 (0.56 to 0.71)	0.86 (0.77 to 0.97)
From diagnosed with COVID-19 to death	Type 2 diabetes	5.88 (5.36 to 6.44)	1.42 (1.29 to 1.56)	8.42 (5.91 to 12.00)	2.25 (1.57 to 3.24)	12.24 (7.26 to 20.65)	4.13 (2.42 to 7.05)	1.17 (1.01 to 1.36)	1.28 (1.11 to 1.48)	1.29 (1.13 to 1.47)	1.42 (1.24 to 1.61)
From hospitalised with COVID-19 to death	Charlson - 1:0	3.42 (3.01 to 3.87)	1.67 (1.47 to 1.90)	2.94 (2.19 to 3.94)	2.00 (1.48 to 2.71)	2.11 (1.27 to 3.51)	1.68 (1.01 to 2.79)	1.57 (1.29 to 1.92)	1.43 (1.17 to 1.75)	1.90 (1.53 to 2.35)	1.57 (1.26 to 1.95)
From hospitalised with COVID-19 to death	Charlson - 2:0	3.61 (3.22 to 4.06)	1.46 (1.29 to 1.64)	2.77 (2.03 to 3.77)	1.72 (1.25 to 2.37)	2.71 (1.74 to 4.24)	1.87 (1.19 to 2.94)	1.49 (1.24 to 1.80)	1.33 (1.10 to 1.60)	1.57 (1.28 to 1.92)	1.22 (1.00 to 1.50)
From hospitalised with COVID-19 to death	Charlson - 3+:0	6.46 (5.84 to 7.14)	1.83 (1.64 to 2.05)	5.48 (4.15 to 7.24)	3.22 (2.41 to 4.31)	7.74 (5.21 to 11.48)	4.83 (3.22 to 7.24)	1.88 (1.60 to 2.21)	1.49 (1.26 to 1.76)	2.44 (2.05 to 2.91)	1.63 (1.36 to 1.96)
From hospitalised with COVID-19 to death	Autoimmune condition	1.42 (1.27 to 1.60)	1.19 (1.06 to 1.34)	1.86 (1.34 to 2.57)	1.56 (1.13 to 2.16)	1.39 (0.84 to 2.30)	1.15 (0.69 to 1.90)	1.06 (0.88 to 1.27)	1.08 (0.90 to 1.30)	1.20 (1.00 to 1.44)	1.20 (1.00 to 1.44)
From hospitalised with COVID-19 to death	Chronic kidney disease	3.24 (2.98 to 3.51)	1.25 (1.15 to 1.36)	3.50 (2.56 to 4.79)	2.20 (1.60 to 3.03)	4.88 (3.10 to 7.66)	2.96 (1.87 to 4.69)	1.40 (1.25 to 1.58)	1.19 (1.05 to 1.34)	1.58 (1.38 to 1.81)	1.14 (1.00 to 1.31)
From hospitalised with COVID-19 to death	COPD	2.21 (1.98 to 2.47)	1.14 (1.02 to 1.28)	3.68 (2.69 to 5.02)	2.07 (1.51 to 2.84)	4.14 (2.24 to 7.65)	2.66 (1.44 to 4.93)	1.08 (0.94 to 1.23)	1.05 (0.92 to 1.21)	1.18 (0.90 to 1.55)	1.04 (0.79 to 1.37)
From hospitalised with COVID-19 to death	Dementia	5.48 (4.91 to 6.12)	1.98 (1.77 to 2.22)	NA	NA	NA	NA	2.06 (1.74 to 2.45)	1.64 (1.38 to 1.96)	2.94 (2.52 to 3.43)	2.12 (1.81 to 2.48)
From hospitalised with COVID-19 to death	Heart disease	2.98 (2.77 to 3.22)	1.22 (1.13 to 1.32)	2.81 (2.24 to 3.53)	1.79 (1.42 to 2.27)	3.34 (2.31 to 4.82)	2.13 (1.47 to 3.11)	1.22 (1.09 to 1.36)	1.08 (0.97 to 1.21)	1.50 (1.32 to 1.71)	1.19 (1.05 to 1.36)
From hospitalised with COVID-19 to death	Hyperlipidemia	1.05 (0.95 to 1.16)	1.04 (0.94 to 1.14)	1.16 (0.89 to 1.51)	0.98 (0.75 to 1.27)	1.14 (0.76 to 1.72)	0.76 (0.50 to 1.15)	1.00 (0.87 to 1.15)	1.09 (0.95 to 1.25)	0.94 (0.79 to 1.11)	1.06 (0.89 to 1.25)
From hospitalised with COVID-19 to death	Hypertension	1.43 (1.32 to 1.55)	1.04 (0.96 to 1.13)	1.52 (1.21 to 1.90)	1.06 (0.84 to 1.33)	1.83 (1.30 to 2.57)	1.20 (0.85 to 1.69)	0.99 (0.88 to 1.11)	1.01 (0.90 to 1.14)	1.04 (0.90 to 1.19)	1.05 (0.92 to 1.21)
From hospitalised with COVID-19 to death	Malignant neoplasm	2.24 (2.05 to 2.44)	1.21 (1.11 to 1.33)	2.93 (2.22 to 3.86)	1.80 (1.36 to 2.38)	2.05 (1.32 to 3.17)	1.47 (0.95 to 2.28)	1.27 (1.13 to 1.43)	1.18 (1.05 to 1.33)	1.23 (1.05 to 1.45)	1.13 (0.96 to 1.33)
From hospitalised with COVID-19 to death	Obesity	1.12 (1.04 to 1.21)	1.09 (1.01 to 1.18)	2.06 (1.67 to 2.55)	1.61 (1.30 to 2.00)	1.55 (1.13 to 2.13)	1.37 (1.00 to 1.88)	0.90 (0.80 to 1.01)	1.02 (0.91 to 1.15)	0.84 (0.74 to 0.96)	0.98 (0.85 to 1.11)
From hospitalised with COVID-19 to death	Type 2 diabetes	1.81 (1.66 to 1.97)	1.26 (1.15 to 1.37)	1.70 (1.31 to 2.20)	1.18 (0.91 to 1.53)	3.54 (2.50 to 5.02)	2.52 (1.77 to 3.58)	1.12 (0.99 to 1.26)	1.15 (1.02 to 1.30)	1.26 (1.09 to 1.47)	1.32 (1.13 to 1.53)

Table 20. Estimated hazard ratios for comorbidities: stratified by calendar month

		March		April	
**Transition**	**Variable**	**Unadjusted**	**Adjusted for age and sex**	**Unadjusted**	**Adjusted for age and sex**
From general population to diagnosed with COVID-19	Charlson - 1:0	1.25 (1.21 to 1.29)	1.22 (1.19 to 1.26)	1.33 (1.28 to 1.37)	1.34 (1.29 to 1.39)
From general population to diagnosed with COVID-19	Charlson - 2:0	1.08 (1.05 to 1.12)	1.13 (1.09 to 1.17)	1.11 (1.06 to 1.15)	1.13 (1.09 to 1.18)
From general population to diagnosed with COVID-19	Charlson - 3+:0	1.06 (1.02 to 1.10)	1.32 (1.26 to 1.37)	1.56 (1.50 to 1.62)	1.69 (1.62 to 1.77)
From general population to diagnosed with COVID-19	Autoimmune condition	1.28 (1.24 to 1.32)	1.17 (1.13 to 1.21)	1.30 (1.25 to 1.35)	1.16 (1.11 to 1.20)
From general population to diagnosed with COVID-19	Chronic kidney disease	0.94 (0.90 to 0.99)	1.18 (1.13 to 1.24)	1.32 (1.26 to 1.38)	1.18 (1.12 to 1.24)
From general population to diagnosed with COVID-19	COPD	0.97 (0.92 to 1.03)	1.07 (1.01 to 1.14)	1.23 (1.16 to 1.31)	1.32 (1.24 to 1.41)
From general population to diagnosed with COVID-19	Dementia	1.63 (1.51 to 1.75)	2.07 (1.91 to 2.25)	4.33 (4.09 to 4.57)	4.26 (4.00 to 4.54)
From general population to diagnosed with COVID-19	Heart disease	1.07 (1.04 to 1.10)	1.23 (1.19 to 1.26)	1.21 (1.17 to 1.25)	1.24 (1.19 to 1.28)
From general population to diagnosed with COVID-19	Hyperlipidemia	1.20 (1.17 to 1.23)	1.09 (1.06 to 1.12)	1.17 (1.13 to 1.20)	1.14 (1.10 to 1.18)
From general population to diagnosed with COVID-19	Hypertension	1.12 (1.09 to 1.14)	1.09 (1.06 to 1.11)	1.13 (1.10 to 1.16)	1.12 (1.09 to 1.15)
From general population to diagnosed with COVID-19	Malignant neoplasm	1.04 (1.00 to 1.08)	1.07 (1.03 to 1.11)	1.11 (1.07 to 1.16)	1.07 (1.02 to 1.11)
From general population to diagnosed with COVID-19	Obesity	1.29 (1.26 to 1.31)	1.22 (1.20 to 1.25)	1.29 (1.26 to 1.33)	1.24 (1.21 to 1.27)
From general population to diagnosed with COVID-19	Type 2 diabetes	1.00 (0.97 to 1.04)	1.03 (1.00 to 1.07)	1.11 (1.07 to 1.16)	1.14 (1.09 to 1.18)
From general population to hospitalised with COVID-19	Charlson - 1:0	3.25 (2.99 to 3.53)	1.40 (1.29 to 1.53)	3.34 (2.95 to 3.78)	1.56 (1.37 to 1.77)
From general population to hospitalised with COVID-19	Charlson - 2:0	4.85 (4.50 to 5.23)	1.55 (1.43 to 1.68)	5.27 (4.72 to 5.88)	1.80 (1.60 to 2.03)
From general population to hospitalised with COVID-19	Charlson - 3+:0	8.56 (8.00 to 9.16)	2.12 (1.96 to 2.30)	10.60 (9.63 to 11.66)	2.75 (2.46 to 3.09)
From general population to hospitalised with COVID-19	Autoimmune condition	2.12 (1.94 to 2.32)	1.30 (1.19 to 1.43)	2.32 (2.04 to 2.63)	1.42 (1.25 to 1.61)
From general population to hospitalised with COVID-19	Chronic kidney disease	5.29 (4.92 to 5.68)	1.43 (1.32 to 1.55)	6.47 (5.87 to 7.14)	1.64 (1.47 to 1.83)
From general population to hospitalised with COVID-19	COPD	4.78 (4.36 to 5.24)	1.50 (1.36 to 1.64)	4.50 (3.92 to 5.15)	1.51 (1.31 to 1.73)
From general population to hospitalised with COVID-19	Dementia	5.20 (4.52 to 5.98)	1.33 (1.15 to 1.54)	12.46 (10.83 to 14.33)	2.93 (2.52 to 3.40)
From general population to hospitalised with COVID-19	Heart disease	4.43 (4.19 to 4.69)	1.38 (1.30 to 1.48)	4.85 (4.47 to 5.27)	1.51 (1.38 to 1.65)
From general population to hospitalised with COVID-19	Hyperlipidemia	2.41 (2.26 to 2.58)	1.22 (1.14 to 1.30)	1.97 (1.77 to 2.18)	1.05 (0.94 to 1.17)
From general population to hospitalised with COVID-19	Hypertension	3.19 (3.01 to 3.38)	1.15 (1.08 to 1.22)	2.76 (2.54 to 3.01)	1.03 (0.94 to 1.13)
From general population to hospitalised with COVID-19	Malignant neoplasm	3.70 (3.44 to 3.97)	1.26 (1.17 to 1.36)	3.46 (3.11 to 3.85)	1.19 (1.07 to 1.33)
From general population to hospitalised with COVID-19	Obesity	3.58 (3.39 to 3.78)	1.88 (1.78 to 1.99)	2.95 (2.72 to 3.20)	1.59 (1.46 to 1.72)
From general population to hospitalised with COVID-19	Type 2 diabetes	4.26 (3.99 to 4.55)	1.50 (1.40 to 1.61)	4.44 (4.03 to 4.88)	1.65 (1.50 to 1.82)

From diagnosed with COVID-19 to hospitalised with COVID-19	Charlson - 1:0	1.76 (1.63 to 1.90)	1.05 (0.97 to 1.14)	1.89 (1.66 to 2.15)	1.08 (0.94 to 1.23)
From diagnosed with COVID-19 to hospitalised with COVID-19	Charlson - 2:0	2.75 (2.55 to 2.96)	1.20 (1.10 to 1.29)	2.74 (2.42 to 3.11)	1.22 (1.06 to 1.40)
From diagnosed with COVID-19 to hospitalised with COVID-19	Charlson - 3+:0	4.06 (3.77 to 4.37)	1.28 (1.17 to 1.39)	3.74 (3.35 to 4.18)	1.25 (1.09 to 1.43)
From diagnosed with COVID-19 to hospitalised with COVID-19	Autoimmune condition	1.41 (1.29 to 1.54)	1.10 (1.01 to 1.21)	1.37 (1.18 to 1.60)	1.03 (0.88 to 1.19)
From diagnosed with COVID-19 to hospitalised with COVID-19	Chronic kidney disease	3.61 (3.32 to 3.92)	1.23 (1.12 to 1.35)	2.94 (2.58 to 3.34)	1.07 (0.93 to 1.24)
From diagnosed with COVID-19 to hospitalised with COVID-19	COPD	2.77 (2.48 to 3.10)	0.98 (0.87 to 1.10)	2.70 (2.28 to 3.20)	1.11 (0.93 to 1.32)
From diagnosed with COVID-19 to hospitalised with COVID-19	Dementia	2.11 (1.79 to 2.50)	0.61 (0.51 to 0.73)	2.71 (2.32 to 3.18)	0.89 (0.75 to 1.06)
From diagnosed with COVID-19 to hospitalised with COVID-19	Heart disease	2.51 (2.37 to 2.67)	1.05 (0.98 to 1.12)	2.71 (2.46 to 2.98)	1.17 (1.05 to 1.31)
From diagnosed with COVID-19 to hospitalised with COVID-19	Hyperlipidemia	1.86 (1.74 to 1.98)	1.14 (1.07 to 1.21)	1.61 (1.44 to 1.80)	1.04 (0.93 to 1.16)
From diagnosed with COVID-19 to hospitalised with COVID-19	Hypertension	2.31 (2.18 to 2.44)	1.09 (1.03 to 1.16)	2.12 (1.93 to 2.33)	1.06 (0.96 to 1.18)
From diagnosed with COVID-19 to hospitalised with COVID-19	Malignant neoplasm	2.36 (2.18 to 2.55)	1.03 (0.95 to 1.12)	2.36 (2.08 to 2.67)	1.11 (0.97 to 1.26)
From diagnosed with COVID-19 to hospitalised with COVID-19	Obesity	2.30 (2.18 to 2.42)	1.60 (1.52 to 1.68)	2.04 (1.87 to 2.23)	1.51 (1.38 to 1.65)
From diagnosed with COVID-19 to hospitalised with COVID-19	Type 2 diabetes	3.13 (2.92 to 3.35)	1.38 (1.28 to 1.48)	2.96 (2.65 to 3.32)	1.38 (1.22 to 1.55)
From diagnosed with COVID-19 to death	Charlson - 1:0	10.36 (8.47 to 12.67)	2.14 (1.83 to 2.50)	16.95 (14.11 to 20.36)	2.60 (2.15 to 3.14)
From diagnosed with COVID-19 to death	Charlson - 2:0	16.64 (13.69 to 20.22)	1.78 (1.54 to 2.06)	17.25 (14.20 to 20.96)	1.59 (1.30 to 1.94)
From diagnosed with COVID-19 to death	Charlson - 3+:0	67.34 (56.96 to 79.59)	2.69 (2.39 to 3.02)	56.12 (47.58 to 66.18)	2.41 (2.02 to 2.88)
From diagnosed with COVID-19 to death	Autoimmune condition	1.81 (1.49 to 2.19)	1.08 (0.89 to 1.32)	2.00 (1.70 to 2.36)	1.14 (0.97 to 1.35)
From diagnosed with COVID-19 to death	Chronic kidney disease	24.10 (21.34 to 27.23)	5.92 (5.26 to 6.66)	13.26 (11.88 to 14.80)	1.07 (0.96 to 1.20)
From diagnosed with COVID-19 to death	COPD	7.59 (6.31 to 9.12)	1.18 (0.98 to 1.42)	4.60 (3.85 to 5.49)	0.97 (0.81 to 1.17)
From diagnosed with COVID-19 to death	Dementia	61.04 (53.62 to 69.48)	2.61 (2.27 to 3.00)	40.35 (36.33 to 44.81)	2.86 (2.56 to 3.19)
From diagnosed with COVID-19 to death	Heart disease	10.07 (8.96 to 11.32)	1.09 (0.97 to 1.22)	6.97 (6.28 to 7.74)	0.85 (0.76 to 0.94)
From diagnosed with COVID-19 to death	Hyperlipidemia	1.60 (1.36 to 1.88)	1.08 (0.92 to 1.28)	1.52 (1.31 to 1.75)	0.95 (0.82 to 1.10)
From diagnosed with COVID-19 to death	Hypertension	3.85 (3.41 to 4.35)	1.01 (0.90 to 1.15)	3.76 (3.37 to 4.19)	1.03 (0.93 to 1.15)
From diagnosed with COVID-19 to death	Malignant neoplasm	6.28 (5.47 to 7.21)	1.10 (0.96 to 1.26)	5.42 (4.79 to 6.14)	1.18 (1.04 to 1.33)
From diagnosed with COVID-19 to death	Obesity	1.99 (1.75 to 2.26)	1.05 (0.92 to 1.19)	1.27 (1.12 to 1.43)	0.79 (0.70 to 0.89)
From diagnosed with COVID-19 to death	Type 2 diabetes	6.60 (5.76 to 7.57)	1.57 (1.37 to 1.80)	5.08 (4.48 to 5.75)	1.28 (1.13 to 1.45)
From hospitalised with COVID-19 to death	Charlson - 1:0	2.83 (2.43 to 3.29)	1.49 (1.28 to 1.74)	5.25 (4.18 to 6.60)	2.01 (1.59 to 2.55)
From hospitalised with COVID-19 to death	Charlson - 2:0	3.18 (2.77 to 3.64)	1.44 (1.24 to 1.66)	5.03 (4.04 to 6.26)	1.46 (1.16 to 1.85)
From hospitalised with COVID-19 to death	Charlson - 3+:0	5.77 (5.13 to 6.49)	1.85 (1.62 to 2.10)	8.46 (6.98 to 10.24)	1.80 (1.45 to 2.22)
From hospitalised with COVID-19 to death	Autoimmune condition	1.35 (1.17 to 1.56)	1.16 (1.01 to 1.34)	1.57 (1.29 to 1.90)	1.21 (1.00 to 1.48)

From hospitalised with COVID-19 to death	Chronic kidney disease	3.17 (2.87 to 3.50)	1.37 (1.23 to 1.52)	3.41 (2.95 to 3.94)	1.05 (0.91 to 1.22)
From hospitalised with COVID-19 to death	COPD	2.39 (2.10 to 2.72)	1.22 (1.07 to 1.39)	1.87 (1.50 to 2.32)	0.99 (0.79 to 1.23)
From hospitalised with COVID-19 to death	Dementia	4.36 (3.71 to 5.11)	1.70 (1.44 to 2.01)	6.52 (5.56 to 7.64)	2.07 (1.76 to 2.44)
From hospitalised with COVID-19 to death	Heart disease	2.82 (2.57 to 3.09)	1.25 (1.14 to 1.38)	3.39 (2.96 to 3.88)	1.13 (0.98 to 1.30)
From hospitalised with COVID-19 to death	Hyperlipidemia	1.11 (0.99 to 1.25)	1.08 (0.96 to 1.21)	0.94 (0.78 to 1.13)	0.96 (0.80 to 1.16)
From hospitalised with COVID-19 to death	Hypertension	1.39 (1.27 to 1.53)	1.04 (0.94 to 1.14)	1.56 (1.35 to 1.80)	1.05 (0.91 to 1.21)
From hospitalised with COVID-19 to death	Malignant neoplasm	2.29 (2.06 to 2.54)	1.30 (1.16 to 1.44)	2.18 (1.86 to 2.55)	1.09 (0.92 to 1.28)
From hospitalised with COVID-19 to death	Obesity	1.18 (1.08 to 1.30)	1.19 (1.08 to 1.30)	1.02 (0.89 to 1.18)	0.92 (0.79 to 1.06)
From hospitalised with COVID-19 to death	Type 2 diabetes	1.81 (1.63 to 2.01)	1.27 (1.15 to 1.41)	1.79 (1.54 to 2.09)	1.22 (1.05 to 1.43)

Figure 3. Comorbidities and COVID-19 transitions: stratified by calendar month (adjusted for age and sex)

Estimated hazard ratios for comorbidities of interest from cause-specific Cox models for each transition. Points give estimated hazard ratios, with lines representing 95% confidence intervals. Models were estimated separately for each comorbidity of interest, with adjustment for age and sex.

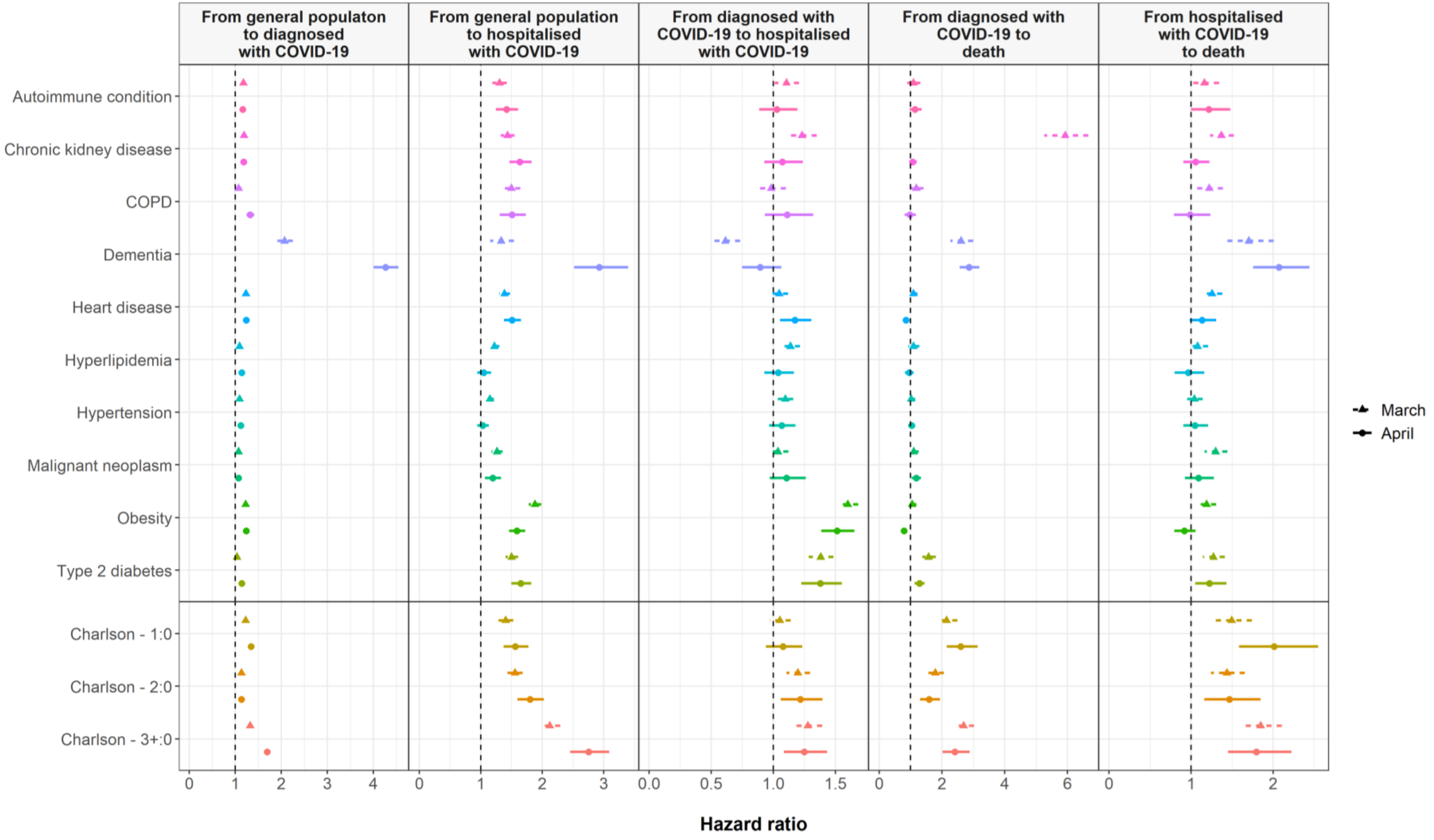

Table 21. Estimated hazard ratios for comorbidities: stratified by region

		Barcelona		Girona		Lleida		Tarragona	
**Transition**	**Variable**	**Unadjusted**	**Adjusted for age and sex**	**Unadjusted**	**Adjusted for age and sex**	**Unadjusted**	**Adjusted for age and sex**	**Unadjusted**	**Adjusted for age and sex**
From general population to diagnosed with COVID-19	Charlson - 1:0	1.30 (1.27 to 1.33)	1.26 (1.23 to 1.30)	1.38 (1.26 to 1.51)	1.52 (1.38 to 1.66)	1.23 (1.14 to 1.32)	1.30 (1.21 to 1.40)	1.12 (1.00 to 1.26)	1.20 (1.06 to 1.35)
From general population to diagnosed with COVID-19	Charlson - 2:0	1.10 (1.07 to 1.13)	1.11 (1.08 to 1.14)	1.02 (0.92 to 1.14)	1.26 (1.12 to 1.41)	1.03 (0.95 to 1.12)	1.18 (1.08 to 1.28)	0.98 (0.86 to 1.12)	1.17 (1.02 to 1.36)
From general population to diagnosed with COVID-19	Charlson - 3+:0	1.25 (1.21 to 1.29)	1.42 (1.37 to 1.47)	1.17 (1.03 to 1.32)	1.62 (1.41 to 1.86)	1.35 (1.24 to 1.48)	1.75 (1.58 to 1.93)	1.26 (1.09 to 1.45)	1.74 (1.48 to 2.04)
From general population to diagnosed with COVID-19	Autoimmune condition	1.30 (1.26 to 1.33)	1.17 (1.14 to 1.21)	1.16 (1.03 to 1.29)	1.14 (1.02 to 1.28)	1.25 (1.16 to 1.36)	1.17 (1.08 to 1.27)	1.32 (1.16 to 1.50)	1.28 (1.13 to 1.46)
From general population to diagnosed with COVID-19	Chronic kidney disease	1.07 (1.04 to 1.11)	1.15 (1.10 to 1.19)	0.93 (0.80 to 1.08)	1.11 (0.94 to 1.32)	1.07 (0.96 to 1.19)	1.24 (1.10 to 1.40)	1.11 (0.93 to 1.33)	1.43 (1.17 to 1.75)
From general population to diagnosed with COVID-19	COPD	1.05 (1.00 to 1.10)	1.12 (1.07 to 1.18)	1.36 (1.17 to 1.57)	1.63 (1.40 to 1.91)	1.16 (1.01 to 1.33)	1.37 (1.19 to 1.58)	1.39 (1.14 to 1.69)	1.70 (1.38 to 2.10)
From general population to diagnosed with COVID-19	Dementia	2.68 (2.55 to 2.81)	3.10 (2.94 to 3.28)	2.31 (1.91 to 2.79)	3.08 (2.50 to 3.80)	6.33 (5.70 to 7.03)	3.58 (3.03 to 4.22)	1.98 (1.53 to 2.56)	2.49 (1.88 to 3.31)
From general population to diagnosed with COVID-19	Heart disease	1.13 (1.11 to 1.16)	1.21 (1.18 to 1.24)	1.01 (0.93 to 1.11)	1.22 (1.10 to 1.34)	1.07 (1.00 to 1.14)	1.23 (1.14 to 1.32)	1.18 (1.07 to 1.31)	1.49 (1.33 to 1.67)
From general population to diagnosed with COVID-19	Hyperlipidemia	1.19 (1.16 to 1.21)	1.09 (1.06 to 1.11)	1.06 (0.97 to 1.16)	1.12 (1.02 to 1.23)	0.99 (0.92 to 1.07)	0.99 (0.92 to 1.07)	1.08 (0.96 to 1.22)	1.14 (1.01 to 1.29)
From general population to diagnosed with COVID-19	Hypertension	1.15 (1.13 to 1.17)	1.10 (1.08 to 1.13)	0.99 (0.92 to 1.07)	1.11 (1.02 to 1.21)	1.00 (0.94 to 1.06)	1.06 (0.99 to 1.14)	1.00 (0.91 to 1.10)	1.13 (1.02 to 1.26)
From general population to diagnosed with COVID-19	Malignant neoplasm	1.06 (1.03 to 1.09)	1.04 (1.00 to 1.07)	0.97 (0.86 to 1.09)	1.10 (0.97 to 1.25)	1.12 (1.02 to 1.22)	1.19 (1.09 to 1.30)	0.99 (0.86 to 1.15)	1.13 (0.97 to 1.31)
From general population to diagnosed with COVID-19	Obesity	1.33 (1.31 to 1.35)	1.26 (1.23 to 1.28)	1.16 (1.08 to 1.24)	1.19 (1.11 to 1.27)	1.27 (1.21 to 1.34)	1.27 (1.20 to 1.33)	1.04 (0.96 to 1.12)	1.04 (0.95 to 1.13)
From general population to diagnosed with COVID-19	Type 2 diabetes	1.06 (1.03 to 1.09)	1.07 (1.04 to 1.10)	0.92 (0.82 to 1.03)	1.07 (0.95 to 1.20)	1.03 (0.95 to 1.13)	1.15 (1.05 to 1.26)	1.02 (0.89 to 1.16)	1.21 (1.05 to 1.40)
From general population to hospitalised with COVID-19	Charlson - 1:0	3.28 (3.05 to 3.53)	1.43 (1.33 to 1.54)	3.67 (2.42 to 5.58)	1.53 (0.99 to 2.35)	2.89 (2.12 to 3.94)	1.42 (1.03 to 1.96)	3.99 (2.66 to 5.99)	1.82 (1.20 to 2.78)
From general population to	Charlson - 2:0	4.88 (4.57 to	1.58 (1.48 to	5.24 (3.57 to	1.57 (1.04 to	5.75 (4.46 to	2.02 (1.52 to	4.59 (3.03 to	1.47 (0.94 to

hospitalised with COVID-19		5.21)	1.70)	7.70)	2.38)	7.42)	2.67)	6.95)	2.30)
From general population to hospitalised with COVID-19	Charlson - 3+:0	8.93 (8.43 to 9.46)	2.23 (2.08 to 2.39)	10.80 (7.72 to 15.12)	2.32 (1.57 to 3.44)	9.80 (7.69 to 12.47)	2.52 (1.89 to 3.36)	10.55 (7.41 to 15.01)	2.49 (1.65 to 3.76)
From general population to hospitalised with COVID-19	Autoimmune condition	2.19 (2.02 to 2.36)	1.36 (1.26 to 1.47)	3.39 (2.34 to 4.92)	2.03 (1.40 to 2.96)	2.02 (1.48 to 2.77)	1.20 (0.87 to 1.65)	1.96 (1.22 to 3.14)	1.19 (0.74 to 1.91)
From general population to hospitalised with COVID-19	Chronic kidney disease	5.50 (5.17 to 5.84)	1.46 (1.37 to 1.56)	5.36 (3.70 to 7.78)	1.20 (0.80 to 1.79)	6.59 (5.17 to 8.41)	1.62 (1.23 to 2.13)	6.59 (4.54 to 9.59)	1.59 (1.06 to 2.40)
From general population to hospitalised with COVID-19	COPD	4.63 (4.27 to 5.01)	1.49 (1.37 to 1.61)	4.68 (3.01 to 7.27)	1.36 (0.86 to 2.13)	3.80 (2.59 to 5.58)	1.20 (0.81 to 1.78)	6.76 (4.38 to 10.43)	2.11 (1.35 to 3.30)
From general population to hospitalised with COVID-19	Dementia	7.62 (6.88 to 8.44)	1.91 (1.72 to 2.13)	6.46 (3.53 to 11.81)	1.40 (0.75 to 2.63)	8.19 (5.24 to 12.82)	1.82 (1.14 to 2.90)	5.28 (2.60 to 10.71)	1.15 (0.56 to 2.39)
From general population to hospitalised with COVID-19	Heart disease	4.47 (4.25 to 4.69)	1.39 (1.32 to 1.47)	6.17 (4.68 to 8.13)	1.78 (1.30 to 2.43)	4.78 (3.91 to 5.86)	1.46 (1.16 to 1.83)	5.29 (3.94 to 7.10)	1.60 (1.15 to 2.22)
From general population to hospitalised with COVID-19	Hyperlipidemia	2.18 (2.06 to 2.32)	1.11 (1.05 to 1.18)	2.12 (1.47 to 3.04)	1.18 (0.82 to 1.71)	2.16 (1.65 to 2.83)	1.16 (0.88 to 1.52)	2.16 (1.46 to 3.21)	1.26 (0.84 to 1.87)
From general population to hospitalised with COVID-19	Hypertension	3.06 (2.91 to 3.22)	1.11 (1.05 to 1.17)	3.40 (2.56 to 4.52)	1.22 (0.91 to 1.63)	2.97 (2.41 to 3.66)	1.10 (0.89 to 1.37)	2.70 (1.97 to 3.68)	1.03 (0.75 to 1.42)
From general population to hospitalised with COVID-19	Malignant neoplasm	9.67 (9.22 to 10.15)	1.23 (1.16 to 1.31)	9.50 (7.14 to 12.65)	1.10 (0.75 to 1.62)	3.98 (3.09 to 5.13)	1.38 (1.06 to 1.80)	2.65 (1.73 to 4.06)	0.86 (0.55 to 1.33)
From general population to hospitalised with COVID-19	Obesity	3.45 (3.29 to 3.61)	1.82 (1.73 to 1.91)	3.13 (2.37 to 4.12)	1.70 (1.29 to 2.26)	3.51 (2.90 to 4.26)	2.00 (1.64 to 2.44)	2.28 (1.70 to 3.06)	1.23 (0.91 to 1.66)
From general population to hospitalised with COVID-19	Type 2 diabetes	4.36 (4.11 to 4.61)	1.55 (1.47 to 1.65)	3.77 (2.69 to 5.28)	1.29 (0.92 to 1.83)	4.09 (3.21 to 5.21)	1.53 (1.20 to 1.97)	3.47 (2.41 to 5.00)	1.29 (0.89 to 1.87)
From diagnosed with COVID-19 to hospitalised with COVID-19	Charlson - 1:0	1.78 (1.66 to 1.92)	1.04 (0.97 to 1.12)	1.55 (1.11 to 2.18)	0.83 (0.59 to 1.17)	1.86 (1.45 to 2.38)	1.15 (0.89 to 1.49)	1.50 (0.94 to 2.40)	0.80 (0.50 to 1.31)
From diagnosed with COVID-19 to hospitalised with COVID-19	Charlson - 2:0	2.63 (2.45 to 2.81)	1.16 (1.08 to 1.25)	3.18 (2.35 to 4.31)	1.17 (0.84 to 1.62)	3.53 (2.83 to 4.41)	1.63 (1.28 to 2.07)	2.50 (1.61 to 3.90)	0.98 (0.61 to 1.57)
From diagnosed with COVID-19 to hospitalised with COVID-19	Charlson - 3+:0	3.51 (3.29 to 3.75)	1.17 (1.08 to 1.26)	6.07 (4.67 to 7.90)	1.74 (1.25 to 2.43)	4.06 (3.25 to 5.08)	1.39 (1.06 to 1.83)	4.02 (2.73 to 5.92)	1.10 (0.70 to 1.75)
From diagnosed with COVID-19 to hospitalised with COVID-19	Autoimmune condition	1.36 (1.25 to 1.48)	1.07 (0.98 to 1.16)	1.74 (1.24 to 2.44)	1.17 (0.83 to 1.65)	1.55 (1.19 to 2.02)	1.11 (0.85 to 1.45)	1.88 (1.23 to 2.87)	1.27 (0.83 to 1.96)
From diagnosed with COVID-19 to hospitalised with COVID-19	Chronic kidney disease	3.10 (2.87 to 3.34)	1.18 (1.08 to 1.28)	4.31 (3.13 to 5.93)	1.40 (0.97 to 2.00)	3.47 (2.69 to 4.48)	1.20 (0.90 to 1.59)	2.91 (1.77 to 4.78)	0.90 (0.52 to 1.55)
From diagnosed with COVID-19	COPD	2.61 (2.36 to	0.99 (0.89 to	2.85 (1.96 to	0.96 (0.65 to	2.51 (1.74 to	0.96 (0.66 to	7.22 (4.90 to	1.02 (0.58 to

to hospitalised with COVID-19		2.89)	1.10)	4.13)	1.41)	3.62)	1.40)	10.62)	1.79)
From diagnosed with COVID-19 to hospitalised with COVID-19	Dementia	1.95 (1.72 to 2.21)	0.65 (0.57 to 0.74)	1.83 (1.06 to 3.17)	0.50 (0.28 to 0.91)	3.33 (2.34 to 4.75)	1.07 (0.72 to 1.59)	1.04 (0.33 to 3.24)	0.30 (0.093 to 0.96)
From diagnosed with COVID-19 to hospitalised with COVID-19	Heart disease	2.43 (2.30 to 2.56)	1.05 (0.99 to 1.11)	3.86 (3.08 to 4.82)	1.56 (1.22 to 1.99)	2.56 (2.12 to 3.10)	1.03 (0.83 to 1.29)	3.06 (2.23 to 4.18)	1.21 (0.85 to 1.73)
From diagnosed with COVID-19 to hospitalised with COVID-19	Hyperlipidemia	1.79 (1.69 to 1.90)	1.11 (1.04 to 1.18)	1.84 (1.39 to 2.42)	1.13 (0.86 to 1.50)	1.37 (1.05 to 1.78)	0.84 (0.64 to 1.09)	1.47 (0.95 to 2.27)	0.84 (0.54 to 1.30)
From diagnosed with COVID-19 to hospitalised with COVID-19	Hypertension	2.23 (2.11 to 2.34)	1.09 (1.03 to 1.15)	2.20 (1.75 to 2.78)	0.92 (0.72 to 1.17)	2.32 (1.94 to 2.79)	1.09 (0.90 to 1.32)	2.33 (1.70 to 3.20)	0.96 (0.69 to 1.34)
From diagnosed with COVID-19 to hospitalised with COVID-19	Malignant neoplasm	2.25 (2.10 to 2.42)	1.04 (0.96 to 1.12)	2.85 (2.10 to 3.88)	1.02 (0.74 to 1.41)	2.71 (2.15 to 3.41)	1.26 (0.99 to 1.60)	2.66 (1.74 to 4.07)	1.13 (0.73 to 1.76)
From diagnosed with COVID-19 to hospitalised with COVID-19	Obesity	2.20 (2.10 to 2.31)	1.57 (1.50 to 1.65)	2.19 (1.78 to 2.70)	1.38 (1.11 to 1.70)	2.24 (1.91 to 2.63)	1.70 (1.44 to 2.00)	2.34 (1.76 to 3.12)	1.52 (1.14 to 2.04)
From diagnosed with COVID-19 to hospitalised with COVID-19	Type 2 diabetes	2.89 (2.71 to 3.09)	1.30 (1.22 to 1.39)	3.69 (2.82 to 4.81)	1.34 (1.01 to 1.77)	4.16 (3.40 to 5.09)	2.00 (1.62 to 2.47)	2.86 (1.94 to 4.21)	1.09 (0.73 to 1.63)
From diagnosed with COVID-19 to death	Charlson - 1:0	14.02 (12.10 to 16.23)	2.49 (2.14 to 2.90)	15.45 (7.55 to 31.60)	2.24 (1.08 to 4.67)	14.23 (8.91 to 22.73)	2.87 (1.77 to 4.64)	9.38 (5.12 to 17.18)	1.49 (0.79 to 2.80)
From diagnosed with COVID-19 to death	Charlson - 2:0	16.86 (14.50 to 19.61)	1.66 (1.42 to 1.95)	29.54 (14.70 to 59.37)	2.55 (1.25 to 5.21)	17.42 (10.66 to 28.46)	1.88 (1.13 to 3.11)	13.29 (7.19 to 24.55)	1.13 (0.60 to 2.15)
From diagnosed with COVID-19 to death	Charlson - 3+:0	65.01 (57.17 to 73.93)	2.66 (2.31 to 3.06)	101.52 (54.22 to 190.09)	2.96 (1.52 to 5.77)	74.80 (49.95 to 112.02)	2.81 (1.82 to 4.32)	27.34 (15.82 to 47.25)	1.04 (0.57 to 1.87)
From diagnosed with COVID-19 to death	Autoimmune condition	1.77 (1.54 to 2.04)	1.07 (0.93 to 1.23)	3.31 (1.97 to 5.55)	1.71 (1.02 to 2.88)	2.69 (1.85 to 3.92)	1.35 (0.92 to 1.96)	1.77 (0.92 to 3.41)	0.97 (0.50 to 1.87)
From diagnosed with COVID-19 to death	Chronic kidney disease	17.74 (16.22 to 19.39)	1.22 (1.11 to 1.33)	21.88 (14.65 to 32.67)	1.28 (0.86 to 1.91)	26.26 (19.98 to 34.51)	1.65 (1.25 to 2.19)	14.38 (9.00 to 22.96)	0.83 (0.51 to 1.35)
From diagnosed with COVID-19 to death	COPD	5.79 (5.02 to 6.68)	1.07 (0.92 to 1.24)	5.82 (3.32 to 10.21)	0.95 (0.53 to 1.70)	6.46 (4.12 to 10.12)	1.03 (0.65 to 1.63)	10.78 (6.37 to 18.26)	1.44 (0.84 to 2.47)
From diagnosed with COVID-19 to death	Dementia	56.40 (51.63 to 61.61)	3.04 (2.77 to 3.34)	43.20 (29.26 to 63.77)	1.93 (1.29 to 2.90)	43.72 (32.81 to 58.24)	2.03 (1.50 to 2.75)	38.98 (24.75 to 61.40)	2.30 (1.44 to 3.66)
From diagnosed with COVID-19 to death	Heart disease	8.31 (7.64 to 9.05)	0.96 (0.88 to 1.04)	7.61 (5.18 to 11.19)	1.03 (0.70 to 1.53)	9.39 (7.19 to 12.27)	0.84 (0.63 to 1.11)	10.55 (7.01 to 15.89)	1.06 (0.69 to 1.62)
From diagnosed with COVID-19 to death	Hyperlipidemia	1.59 (1.41 to 1.78)	1.04 (0.93 to 1.17)	1.96 (1.17 to 3.29)	0.97 (0.58 to 1.63)	0.98 (0.58 to 1.66)	0.63 (0.37 to 1.06)	1.16 (0.56 to 2.40)	1.06 (0.50 to 2.23)
From diagnosed with COVID-19	Hypertension	3.80 (3.48 to	1.05 (0.96 to	3.79 (2.55 to	0.84 (0.56 to	3.67 (2.75 to	0.83 (0.62 to	4.31 (2.81 to	0.91 (0.59 to

to death		4.15)	1.15)	5.64)	1.25)	4.89)	1.11)	6.60)	1.40)
From diagnosed with COVID-19 to death	Malignant neoplasm	5.76 (5.20 to 6.36)	1.13 (1.02 to 1.25)	6.60 (4.19 to 10.39)	0.94 (0.58 to 1.50)	7.61 (5.64 to 10.29)	1.50 (1.11 to 2.04)	4.08 (2.35 to 7.10)	0.67 (0.38 to 1.17)
From diagnosed with COVID-19 to death	Obesity	1.66 (1.51 to 1.83)	0.93 (0.85 to 1.03)	1.33 (0.85 to 2.08)	0.72 (0.46 to 1.15)	1.10 (0.79 to 1.54)	0.82 (0.59 to 1.14)	1.24 (0.75 to 2.03)	0.65 (0.39 to 1.07)
From diagnosed with COVID-19 to death	Type 2 diabetes	5.69 (5.14 to 6.29)	1.38 (1.24 to 1.52)	8.27 (5.45 to 12.56)	1.57 (1.03 to 2.39)	6.08 (4.40 to 8.40)	1.83 (1.31 to 2.54)	7.04 (4.44 to 11.15)	1.51 (0.95 to 2.41)
From hospitalised with COVID-19 to death	Charlson - 1:0	3.41 (2.99 to 3.89)	1.64 (1.43 to 1.88)	3.11 (1.51 to 6.41)	1.82 (0.86 to 3.84)	3.36 (1.98 to 5.68)	1.67 (0.98 to 2.86)	4.98 (2.02 to 12.27)	3.44 (1.35 to 8.76)
From hospitalised with COVID-19 to death	Charlson - 2:0	3.61 (3.19 to 4.08)	1.42 (1.25 to 1.61)	4.14 (2.21 to 7.77)	2.16 (1.11 to 4.21)	3.34 (2.08 to 5.38)	1.43 (0.88 to 2.35)	3.60 (1.34 to 9.68)	2.04 (0.71 to 5.83)
From hospitalised with COVID-19 to death	Charlson - 3+:0	6.40 (5.75 to 7.11)	1.76 (1.57 to 1.98)	7.22 (4.20 to 12.40)	2.86 (1.55 to 5.29)	6.97 (4.63 to 10.50)	1.90 (1.22 to 2.97)	6.42 (2.90 to 14.20)	3.55 (1.45 to 8.69)
From hospitalised with COVID-19 to death	Autoimmune condition	1.49 (1.32 to 1.68)	1.23 (1.09 to 1.39)	1.05 (0.56 to 1.97)	0.88 (0.46 to 1.65)	1.19 (0.73 to 1.95)	1.09 (0.66 to 1.78)	0.36 (0.088 to 1.51)	0.34 (0.082 to 1.40)
From hospitalised with COVID-19 to death	Chronic kidney disease	3.20 (2.93 to 3.49)	1.24 (1.13 to 1.36)	4.76 (3.07 to 7.38)	1.78 (1.07 to 2.96)	3.10 (2.21 to 4.35)	1.09 (0.76 to 1.57)	3.28 (1.72 to 6.25)	1.57 (0.76 to 3.24)
From hospitalised with COVID-19 to death	COPD	2.24 (1.99 to 2.52)	1.14 (1.02 to 1.29)	2.33 (1.36 to 3.99)	1.40 (0.81 to 2.43)	1.32 (0.70 to 2.51)	0.64 (0.33 to 1.22)	3.03 (1.50 to 6.12)	2.06 (1.01 to 4.21)
From hospitalised with COVID-19 to death	Dementia	5.70 (5.08 to 6.39)	2.03 (1.81 to 2.29)	2.32 (1.07 to 5.02)	0.91 (0.41 to 2.01)	4.27 (2.66 to 6.83)	1.41 (0.87 to 2.28)	5.23 (1.86 to 14.68)	3.19 (1.12 to 9.08)
From hospitalised with COVID-19 to death	Heart disease	3.06 (2.82 to 3.31)	1.26 (1.15 to 1.37)	1.97 (1.31 to 2.96)	0.86 (0.55 to 1.35)	2.97 (2.17 to 4.07)	1.02 (0.73 to 1.44)	2.38 (1.32 to 4.26)	1.35 (0.71 to 2.55)
From hospitalised with COVID-19 to death	Hyperlipidemia	1.06 (0.96 to 1.17)	1.05 (0.95 to 1.16)	0.52 (0.26 to 1.04)	0.47 (0.24 to 0.94)	1.24 (0.79 to 1.94)	1.20 (0.76 to 1.89)	1.25 (0.56 to 2.80)	1.30 (0.57 to 2.95)
From hospitalised with COVID-19 to death	Hypertension	1.44 (1.32 to 1.57)	1.05 (0.97 to 1.15)	0.89 (0.56 to 1.41)	0.69 (0.43 to 1.10)	1.59 (1.15 to 2.21)	1.12 (0.81 to 1.57)	1.37 (0.74 to 2.54)	0.95 (0.50 to 1.78)
From hospitalised with COVID-19 to death	Malignant neoplasm	2.21 (2.02 to 2.43)	1.18 (1.07 to 1.30)	2.37 (1.47 to 3.82)	1.61 (0.99 to 2.62)	2.54 (1.80 to 3.60)	1.47 (1.03 to 2.09)	2.07 (1.03 to 4.18)	1.31 (0.63 to 2.73)
From hospitalised with COVID-19 to death	Obesity	1.11 (1.02 to 1.21)	1.08 (0.99 to 1.17)	1.08 (0.71 to 1.65)	1.05 (0.68 to 1.62)	1.29 (0.94 to 1.76)	1.36 (0.99 to 1.87)	1.27 (0.70 to 2.31)	1.19 (0.65 to 2.16)
From hospitalised with COVID-19 to death	Type 2 diabetes	1.79 (1.63 to 1.96)	1.24 (1.13 to 1.36)	2.07 (1.32 to 3.23)	1.29 (0.81 to 2.03)	1.74 (1.23 to 2.47)	1.35 (0.94 to 1.92)	2.48 (1.32 to 4.66)	2.00 (1.05 to 3.80)

Figure 4. Comorbidities and COVID-19 transitions: stratified by region (adjusted for age and sex)

Estimated hazard ratios for comorbidities of interest from cause-specific Cox models for each transition. Points give estimated hazard ratios, with lines representing 95% confidence intervals. Models were estimated separately for each comorbidity of interest, with adjustment for age and sex.

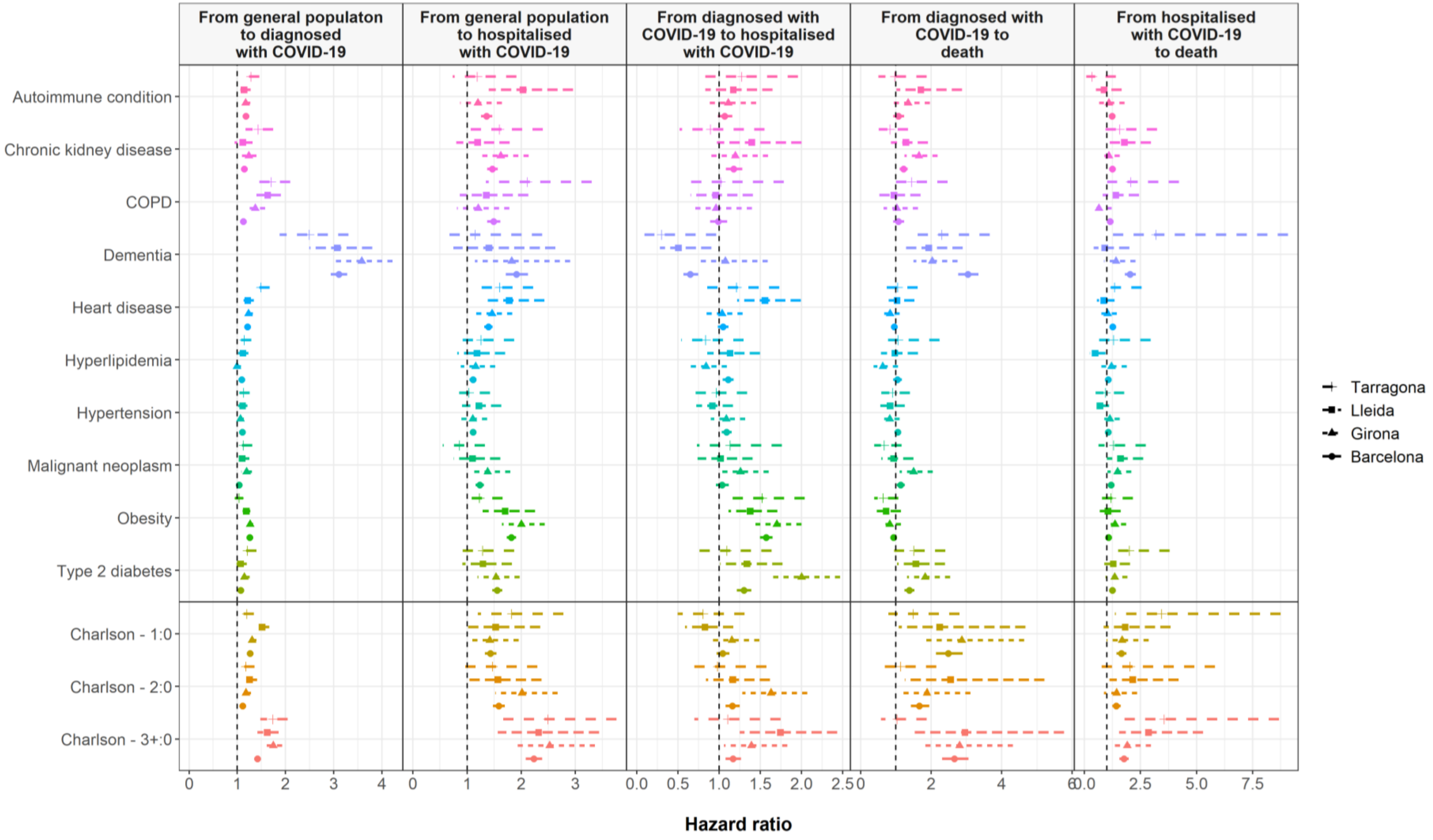

Supplement: Supplementary file 1 — Supplementary Information [file 41467_2021_21100_MOESM1_ESM.pdf]
