# Supplementary material for: The natural history of symptomatic COVID-19 during the first wave in Catalonia
Source: Nat Commun. 2021 Feb 3;12:777. doi: 10.1038/s41467-021-21100-y (PMC7858639; doi:10.1038/s41467-021-21100-y)
Supplement: Supplementary file 3 — Reporting Summary [file 41467_2021_21100_MOESM3_ESM.pdf]

nature research

Corresponding author(s): Daniel Prieto Alhambra

Last updated by author(s): Dec 14, 2020

Reporting Summary

Nature Research wishes to improve the reproducibility of the work that we publish. This form provides structure for consistency and transparency in reporting. For further information on Nature Research policies, see our Editorial Policies and the Editorial Policy Checklist.

Statistics

For all statistical analyses, confirm that the following items are present in the figure legend, table legend, main text, or Methods section.

n/a	Confirmed	
☐	☒	The exact sample size (n) for each experimental group/condition, given as a discrete number and unit of measurement
☒	☐	A statement on whether measurements were taken from distinct samples or whether the same sample was measured repeatedly
☐	☒	The statistical test(s) used AND whether they are one- or two-sided<br>*Only common tests should be described solely by name; describe more complex techniques in the Methods section.*
☐	☒	A description of all covariates tested
☒	☐	A description of any assumptions or corrections, such as tests of normality and adjustment for multiple comparisons
☐	☒	A full description of the statistical parameters including central tendency (e.g. means) or other basic estimates (e.g. regression coefficient) AND variation (e.g. standard deviation) or associated estimates of uncertainty (e.g. confidence intervals)
☐	☒	For null hypothesis testing, the test statistic (e.g. F, t, r) with confidence intervals, effect sizes, degrees of freedom and P value noted<br>*Give P values as exact values whenever suitable.*
☒	☐	For Bayesian analysis, information on the choice of priors and Markov chain Monte Carlo settings
☒	☐	For hierarchical and complex designs, identification of the appropriate level for tests and full reporting of outcomes
☒	☐	Estimates of effect sizes (e.g. Cohen's d, Pearson's r), indicating how they were calculated

Our web collection on statistics for biologists contains articles on many of the points above.

Software and code

Policy information about availability of computer code

Data collection	Individual-level routinely-collected primary care data were extracted from the Information System for Research in Primary Care (SIDIAP; www.sidiap.org) database, which captures patient records from approximately 80% of the Catalan population
Data analysis	The data analysis was performed in R 4.0.0, with the study code used available at https://github.com/SIDIAP/MultiStateCovid-19

For manuscripts utilizing custom algorithms or software that are central to the research but not yet described in published literature, software must be made available to editors and reviewers. We strongly encourage code deposition in a community repository (e.g. GitHub). See the Nature Research guidelines for submitting code & software for further information.

Data

Policy information about availability of data

All manuscripts must include a data availability statement. This statement should provide the following information, where applicable:

- Accession codes, unique identifiers, or web links for publicly available datasets
- A list of figures that have associated raw data
- A description of any restrictions on data availability

In accordance with current European and national law, the data used in this study is only available for the researchers participating in this study. Thus, we are not allowed to distribute or make publicly available the data to other parties. However, researchers from public institutions can request data from SIDIAP if they comply with certain requirements. Further information is available online (https://www.sidiap.org/index.php/menu-solicitudes-en/application-proccedure) or by contacting Anna Moleras (amoleras@idiapjgol.org).

Field-specific reporting

Please select the one below that is the best fit for your research. If you are not sure, read the appropriate sections before making your selection.

☒ Life sciences ☐ Behavioural & social sciences ☐ Ecological, evolutionary & environmental sciences

For a reference copy of the document with all sections, see nature.com/documents/nr-reporting-summary-flat.pdf

Life sciences study design

All studies must disclose on these points even when the disclosure is negative.

Sample size	All individuals who were eligible to be included were included in the study.
Data exclusions	As specified in the ethics application for the study, where less than 10 individuals contributed a particular characteristic this characteristic has not been reported so as to maintain patient confidentiality.
Replication	N/A (this was an observational study of routinely-collected health care data)
Randomization	N/A (this was an observational study of routinely-collected health care data)
Blinding	N/A (this was an observational study of routinely-collected health care data)

Reporting for specific materials, systems and methods

We require information from authors about some types of materials, experimental systems and methods used in many studies. Here, indicate whether each material, system or method listed is relevant to your study. If you are not sure if a list item applies to your research, read the appropriate section before selecting a response.

Materials & experimental systems

n/a	Involved in the study	
☒	☐	Antibodies
☒	☐	Eukaryotic cell lines
☒	☐	Palaeontology and archaeology
☒	☐	Animals and other organisms
☐	☒	Human research participants
☒	☐	Clinical data
☒	☐	Dual use research of concern

Methods

n/a	Involved in the study	
☒	☐	ChIP-seq
☒	☐	Flow cytometry
☒	☐	MRI-based neuroimaging

Human research participants

Policy information about studies involving human research participants

Population characteristics	A population-based cohort study including all individuals registered in Information System for Research in Primary Care (SIDIAP). SIDIAP includes primary care records covering > 80% of the population of Catalonia, Spain, and was linked to region-wide testing, hospital and mortality records.
Recruitment	N/A (This study is descriptive in nature and performed in response to the COVID-19 pandemic)
Ethics oversight	This study was approved by the Clinical Research Ethics Committee of the IDIAPJGol (project code: 20/070-PCV).

Note that full information on the approval of the study protocol must also be provided in the manuscript.
